# Comparative transcriptome analysis of endemic and epidemic Kaposi’s sarcoma (KS) lesions and the secondary role of HIV-1 in KS pathogenesis

**DOI:** 10.1371/journal.ppat.1008681

**Published:** 2020-07-24

**Authors:** Salum J. Lidenge, Andrew V. Kossenkov, For Yue Tso, Jayamanna Wickramasinghe, Sara R. Privatt, Owen Ngalamika, John R. Ngowi, Julius Mwaiselage, Paul M. Lieberman, John T. West, Charles Wood

**Affiliations:** 1 Nebraska Center for Virology and the School of Biological Sciences, University of Nebraska, Lincoln, Nebraska, United States of America; 2 Ocean Road Cancer Institute, Dar es Salaam, Tanzania; 3 Muhimbili University of Health and Allied Sciences, Dar es Salaam, Tanzania; 4 Wistar Institute, Philadelphia, Pennsylvania, United States of America; 5 Dermatology and Venereology section, University Teaching Hospitals, University of Zambia School of Medicine, Lusaka, Zambia; 6 Nebraska Center for Virology and the Department of Biochemistry, University of Nebraska, Lincoln, Nebraska, United States of America; University of South Florida, UNITED STATES

## Abstract

In sub-Saharan Africa, endemic Kaposi’s sarcoma (EnKS) is still prevalent despite high incidence of epidemic Kaposi’s sarcoma (EpKS) resulting from the on-going HIV-1 epidemic. While KSHV is clearly the etiologic agent of KS, the mechanisms underlying KS development are not fully understood. For example, HIV-1 co-infection and concomitant immune dysfunction have been associated with EpKS development. However, the direct or indirect role(s) of HIV-1, and therefore of immune suppression, in EpKS remains unclear. How, or whether, EpKS is mechanistically distinct from EnKS is unknown. Thus, the absence of HIV-1 co-infection in EnKS provides a unique control for investigating and deciphering whether HIV-1 plays a direct or indirect role in the EpKS tumor microenvironment. We hypothesized that HIV-1 co-infection would induce transcriptome changes that differentiate EpKS from EnKS, thereby defining the direct intra-tumor role of HIV-1 in KS. Comparison of ART-treated and -naïve patients would further define the impact of ART on the KS transcriptome. We utilized RNA-seq followed by multiparameter bioinformatics analysis to compare transcriptomes from KS lesions to uninvolved control skin. We provide the first transcriptomic comparison of EpKS versus EnKS, ART-treated vs–naïve EpKS and male vs female EpKS to define the roles of HIV-1 co-infection, the impact of ART, and gender on KS gene expression profiles. Our findings suggest that ART-use and gender have minimal impact on transcriptome profiles of KS lesions. Gene expression profiles strongly correlated between EpKS and EnKS patients (Spearman r = 0.83, p<10^−10^). A subset of genes involved in tumorigenesis and inflammation/immune responses showed higher magnitude, but not unique dysregulation in EnKS compared to EpKS. While gender and ART had no detectable contribution, the trend toward higher magnitude of gene dysregulation in EnKS coupled with the absence of HIV-1 transcripts in EpKS may suggest an indirect or systemic effect of HIV-1 to promote KS tumorigenesis.

## Author summary

Despite improved antiretroviral therapy (ART) coverage, Kaposi’s sarcoma (KS) remains the most common cancer in people living with HIV/AIDS. KSHV is known to cause KS, but there are no preventive or curative vaccines. Our understanding of the interactions between KSHV, host cell and the microenvironment is lacking. HIV-1 co-infection has been implicated in KS pathogenesis, but its mechanistic role is unclear. We analyzed transcriptomes from lesions and uninvolved control skin from HIV-1-positive and -negative KS patients to determine the direct or indirect role of HIV-1 in KS development. Transcriptomes from the uninvolved control skin from KS subjects were indistinguishable from that of non-KS healthy individuals. This validates the use of uninvolved control skin to control for gene expression pattern in KS lesions. Despite high concordance in gene expression profiles between HIV-1-positive and -negative KS patients, a subset of genes involved in tumorigenesis and inflammation/immune responses showed higher magnitude of dysregulation in EnKS than EpKS patients. The trend toward lower magnitude of gene dysregulation in EpKS coupled with the absence of HIV-1-transcripts in EpKS suggests an indirect or systemic effect of HIV-1 to promote KS tumorigenesis and possibly explain the high KS incidence in regions with endemic KSHV and HIV-1 co-infections.

## Introduction

Kaposi’s sarcoma-associated herpesvirus (KSHV) is an oncogenic gamma-herpesvirus associated with Kaposi’s sarcoma (KS), primary effusion lymphoma (PEL) and multicentric Castleman’s disease (MCD) [1–4]. KSHV infection is unequally distributed geographically with endemic infection evident in sub-Saharan Africa (SSA), parts of Eastern Europe, the Mediterranean, and in Uygur ethnicities in Xinjiang, China [5–8]. In SSA, KSHV seroprevalence is generally high, ranging from 30% to 90% [5,9–11]. In the US and much of the developed world, the prevalence of KSHV infection is low in the general population; however, KSHV prevalence is substantially elevated in high-risk groups, such as the HIV-1 infected individuals, and men who have sex with men [12]. Saliva is the major route of KSHV transmission, and in endemic regions most infections occur in childhood before the first five years of life [13–15].

There are four major forms of KS, classical KS, iatrogenic KS, African-endemic KS (EnKS) and epidemic/HIV-1/AIDS-associated KS (EpKS) [16–20]. EnKS and EpKS are the most common forms in SSA. EnKS is typically an indolent, predominantly cutaneous disease in HIV-1 seronegative individuals, although visceral disease can also occur. Before the HIV-1/AIDS epidemic, EnKS caused between 4–10% of sub-Saharan adult cancers [21–23]. Although common in men, EnKS also affects women and even children, in whom it can present as an aggressive disease with lymphoid involvement [16]. Concomitant with the HIV-1/AIDS epidemic in SSA, EpKS surpassed EnKS to become one of the most common cancers in both genders, in whom it causes significant morbidity and mortality in the region [24–27].

Our group and others have previously analyzed viral and cellular gene expression profiles of EpKS patients [28–30]. However, the impact of HIV-1 on transcriptome profiles of KS lesions has not been investigated. While KSHV is clearly the etiologic agent of KS [1], the mechanisms underlying KS development are not fully understood. HIV-1 co-infection and associated immune dysfunction have been epidemiologically associated with EpKS development, but the mechanistic underpinnings of this association remain unclear. Without antiretroviral therapy (ART), nearly 50% of KSHV and HIV-1 co-infected individuals develop KS, consistent with the concept that HIV-1 immunosuppression promotes KS [31–34]. Similarly, chemotherapeutic immune suppression in organ transplantation has been associated with KS development [35–37]. This iatrogenic form of KS resolves when the chemo-suppression is removed [35]. Importantly, transcriptome profile of EnKS patients is unknown as previous studies were performed on EpKS patients. How, or whether, EpKS in the presence of HIV co-infection is mechanistically distinct from EnKS in the disease development has not previously been explored. Thus, the comparison between these two forms of KS will be important in investigating and deciphering the role of HIV-1 in the KS tumor microenvironment and for determining whether it is direct or indirect. Previous reports on immune responses in EnKS are sparse and the reports are conflicting, where one study reported no evidence of immune dysregulations in EnKS [38], while others suggest lower CD4:CD8 T-cell ratios and increased serum immunoglobulin G in EnKS patients versus controls [39,40]. Our recent analyses of EnKS patients showed similar CD4:CD8 ratios between EnKS and healthy individuals, and no significant differences between EnKS and EpKS patients in terms of cytokines and antibody responses [41].

Apart from inducing anti-inflammatory cytokines [41], KSHV is known to induce metabolic changes in infected cells [18,42,43]. Our recent RNA-seq analysis comparing EpKS tumor tissue from ART treated patients to uninvolved control skin from the same individuals, revealed profound dysregulation in glucose metabolic pathways, suggestive of a Warburg-like effect in KS tumors [29]. However, the lipid metabolic activities in KS tumors versus uninvolved control skin were surprisingly low [29]. Whether the same dysregulations, or similar magnitude of changes, occur in EnKS, has not been investigated. Because lipid metabolic dysregulation is common in ART treated HIV-1 infected individuals [44–47], it will be important to determine whether the KS-associated lipid metabolism defects are due to KSHV infection, attributable to HIV-1 co-infection or result from ART. Moreover, a male gender bias in KS has been noted, especially for classical and EnKS, suggesting a potential involvement of androgens or androgen receptors in the disease pathogenesis [48–52]. Importantly, men from SSA are more likely to be KSHV seropositive compared to women [53].

In this study, we provide the first transcriptomic comparison of EpKS vs EnKS patients, ART-treated versus -naïve EpKS patients and male versus female EpKS patients to more completely define the roles of HIV-1 co-infection, ART experience, and gender on KS gene expression profiles. We initially hypothesized that HIV-1 co-infection would induce transcriptome changes that differentiate EpKS from EnKS, thereby defining the direct intra-tumor role of HIV-1 in KS. Comparison of ART-treated and–naïve patients would define the impact of ART on tumor transcriptome. These differentials could prove important in the design of therapeutic strategies to control KSHV and KS development. In addition, we have compared transcriptomes from KS lesions to uninvolved control skin from the same patient as well from asymptomatic volunteers. This is to determine whether KSHV has a systematic effect on gene expression profiles, even in non-disease sites, and to ensure that uninvolved control skin in KS patients is indeed ‘normal’.

Our results demonstrate that there are no differentials in most of the transcriptomes between EpKS and EnKS, however a small subset of genes exhibited a higher magnitude of change in EnKS compared to EpKS. This coupled with the absence of HIV-1 transcripts in EpKS suggest an indirect or systemic effect of HIV-1 to promote cellular reprogramming of this group of genes which may be involved in KS development. The data also demonstrated that ART-use and gender have minimal impact on transcriptome profiles of KS lesions. Furthermore, uninvolved control skin from KS subjects is indistinguishable from that of asymptomatic healthy controls.

## Materials and methods

### Study subjects and sample processing

KS patients of both genders (N = 24), over 18 years of age were recruited from Tanzania and Zambia. Diagnosis of KS was confirmed by histology and PCR detection of KSHV viral DNA in the lesion. For every KS patient recruited in the study, a 4mm skin biopsy was collected from a representative KS lesion. Similarly, another 4mm skin biopsy was collected from an uninvolved control contralateral/ipsilateral cutaneous site from the same individual. However, because it is possible that even uninvolved control skin tissues in KS patients are transcriptomically dysregulated in relation to skin from non-KS subjects, 4mm skin biopsies were collected from three healthy volunteers for comparison of gene expression profiles. Written informed consent was obtained from all study participants. All biopsies were treated with *RNAlater* (Life Technologies, Carlsbad, CA) overnight at 4°C to prevent RNA degradation and then frozen at -80°C after removal of *RNAlater*.

**Fig 1:**
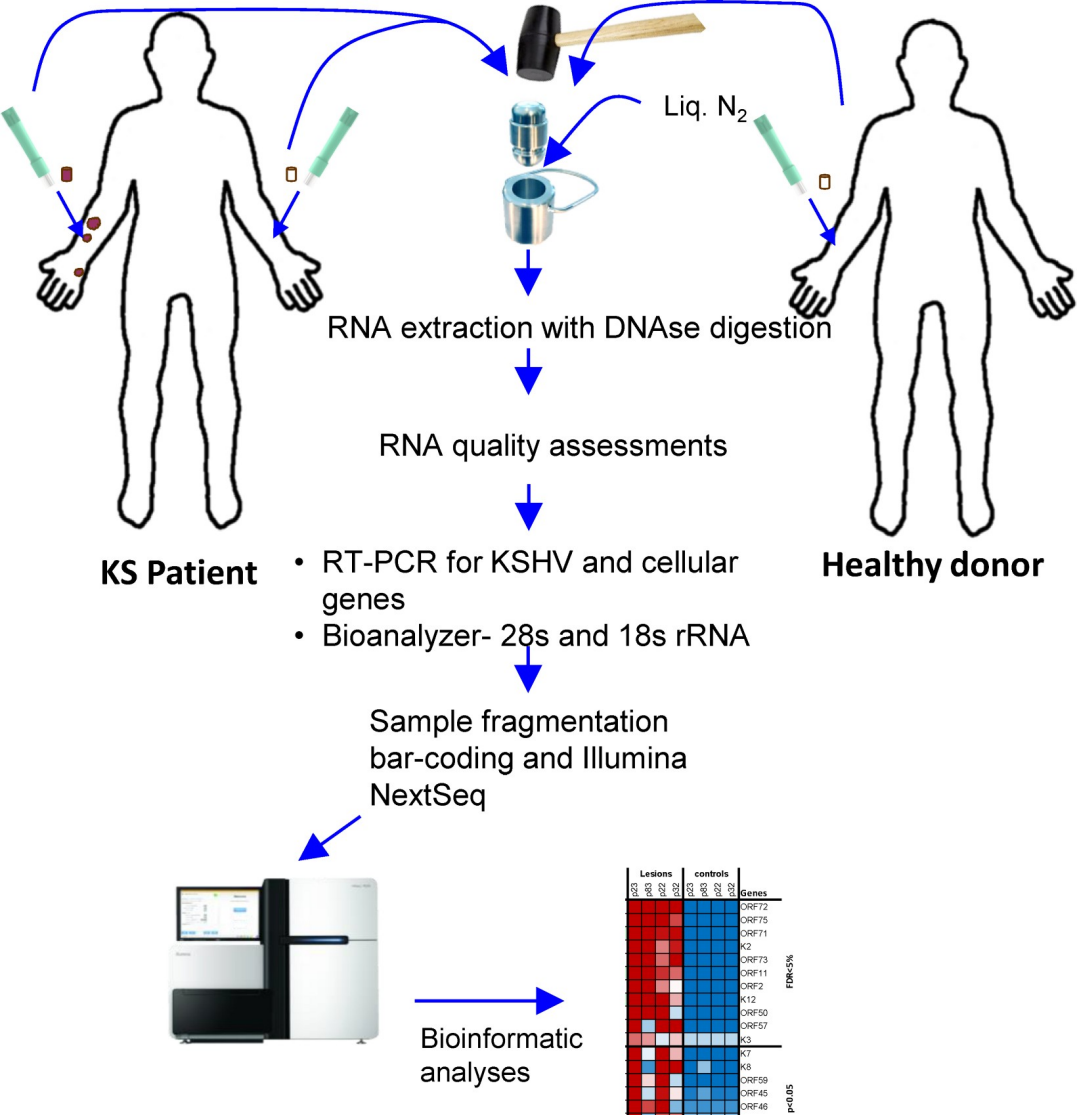
Schematic representation of sample collection, processing, and sequencing. Liq. N_2_ –Liquid nitrogen.

Biopsied tissues were subjected to RNA extraction using a combined cryo-pulverization (mortar and pestle) and miRNeasy Mini Kit (Qiagen, Hilden, Germany) procedure. Briefly, in a metal tray, the DNA/RNA free mortar and pestle were chilled using liquid nitrogen. Biopsied tissue samples were transferred (one at a time) from dry ice into the pre-chilled mortar and then pulverized and homogenized using the pre-chilled pestle. The homogenized sample was then transferred to a 2mL Eppendorf tube using a pre-chilled spatula. The miRNeasy Mini Kit (Qiagen, Hilden, Germany) was used to extract total RNA from homogenized samples using on-column DNase-I treatment, according to the manufacturer’s protocol. RNA concentration was determined using a Qubit fluorimeter (Invitrogen, Massachusetts, USA) and the quality of the extracted RNA was assessed by RNA fragment analysis using an Agilent Bio-analyzer (Agilent Technologies, Santa Clara, USA). The electrophoretic trace of the 18s and 28s rRNA peaks was used to calculate an RNA Integrity Number (RIN) indicative of the level of RNA integrity. All sequenced samples had RIN ranging from 4.8 to 10. An overall schematic representation of biopsy collection and processing is shown in Fig 1.

Plasma was separated from whole blood samples by centrifugation at 400xg for 5 minutes, followed by volume reconstitution and preparation of the peripheral blood mononuclear cell (PBMC) fraction by density gradient centrifugation using Lymphoprep (Stemcell technologies, Massachusetts, USA). Plasma and cells with cryoprotectant were stored at -80°C. The collected plasma was then used to determine the serology and plasma viral load for both HIV-1 and KSHV. Approval to conduct this study was obtained from the review boards of Tanzania National Institute for Medical Research, Ocean Road Cancer Institute, University of Zambia Biomedical Research Ethics Committee, and the University of Nebraska-Lincoln (UNL).

### HIV-1 serology and plasma viral load quantification by real-time PCR (RT-PCR)

HIV-1 serology was determined by HIV Rapid Test Algorithm [54] in Tanzania or Alere Determine HIV-1/2 Ag/Ab Combo test in Zambia. The results were further verified in our lab at Lincoln, Nebraska using HIV-1-2.0 First Response kit (Premier Medical Corporation Limited, Daman, India). To quantify HIV-1 plasma viral load, viral RNA was extracted from the plasma according to the QIAamp viral RNA extraction protocol (Qiagen, Hilden, Germany). Viral copy numbers were determined using RNA Ultra-Sense One-Step quantitative RT-PCR system (Applied Biosystems, Carlsbad, CA) as previously described [55] with universal HIV LTR primers (forward [5’-GCCTCAATAAAGCTTGCCTTGA-3’] and reverse [5’–GGGCGCCACTGCTAGAGA–3’] and probe [5’-FAM/CCAGAGTCACACAACAGACGGGCACA/-BHQ1-3’]) under the following cycling conditions: 50°C for 15 min, 95°C for 2 min, 40 cycles of 95°C for 15 seconds, and 60°C for 30 seconds.

### Plasma KSHV detection and viral load quantification by real-time PCR (RT-PCR)

Plasma samples (400μL) were centrifuged at 8,000 x g at room temperature for 10 minutes to remove any residual cells. To eliminate any cell-free DNA, 15μL of DNase-I (Qiagen, Hilden, Germany) was added and incubated for 2 hours at room temperature. The DNase-I was then inactivated by incubation at 65°C for 20 minutes. KSHV virion-associated DNA (vDNA) was extracted according to the QIAamp DNA extraction protocol (Qiagen, Hilden, Germany). A negative PCR amplification result for the human β-actin gene using primers of Actin1 [5’-TTCTACAATGAGCTGCGTGT-3’] and Actin2 [5’-GCCAGACAGCACTGTGTTGG-3’] demonstrated the complete elimination of any cell-free DNA. KSHV vDNA was then detected by nested PCR of the KSHV ORF26 gene using the following primers, forward [5’-AGCCGAAAGATTCCACCAT-3’] and reverse [5’-TCCGTGTTGTCTACGTCCAG-3’] in the first round, and forward [5’-CGAATCCAACGGATTTGACCTC-3’] and reverse [5’-CCCATAAATGACACATTGGTGGTA-3’] in the second round reaction. Each amplification was performed in a total volume of 25 μl using 0.4 μM primers and Takara Ex Taq DNA polymerase kit (Takara Bio, Mountain View, CA) according to manufacturer’s protocol. 200ng of DNA isolated from plasma was used as a template for both β-actin and KSHV ORF26 in first round amplification. 2 μl of the initial reaction product was used for nested ORF26 second round amplification. All reactions utilized the following thermal-cycling conditions: 95°C for 5 min, 35 cycles of 95°C for 30 secs, 58°C for 30 secs, 72°C for 30 secs, and one cycle of 72°C for 7 min.

### RNA library preparation and sequencing

Biopsy-derived RNA samples with a RIN ranging from 4.8 to 10 were used for library preparation and sequencing. Library preparation was done using the Illumina’s TruSeq RNA Library Prep Kit (San Diego, CA), and data were collected using Illumina’s NextSeq HighOutput v2 (San Diego, CA), in single-read 50bp, rapid run mode at the University of Nebraska DNA Sequencing Core.

### RNA-seq data analysis

The RNA-seq data was processed as previously described [29]. Raw reads were aligned against the human genome and transcriptome (hg19 genome, Ensemble v75 transcriptome) and unaligned reads to KSHV genome and transcriptomes (NC_009333) using the bowtie2 algorithm. RSEM v1.2.31 software was used to estimate read counts and FPKM values at the gene level. Read count normalization (by normalizing KSHV counts to total number of human transcriptomic reads) and significance and fold change of differential expression between lesion and control samples were performed using the DESeq2 algorithm [56] on raw values. Genes with a false discovery rate (FDR) <5% were considered as significant unless stated otherwise. Spearman correlation was used to test associations between KSHV transcript load in tissue (total number of normalized KSHV transcriptomic reads) and normalized gene expression. Principal component analysis (PCA) and hierarchical clustering was performed on DESeq2-normalized log2-scaled and z-score transformed. PCA was used to investigate batch effects on overall transcription measurements. KSHV gene functional definitions were obtained from Arias *et*.*al* [57]. Hierarchical clustering for KSHV genes was done on the expression values normalized to total KSHV transcript load using Spearman correlation distance for genes and Euclidean distance for patients using average linkage. Functions were then assigned for each cluster based on the function with the best enrichment ratio E = % genes in cluster / % genes total. Expression heatmaps were plotted in Microsoft Excel using mean-centered normalized expression values. HLA typing from RNA-seq data was done using OptiType software [58]. Gene set enrichment analysis was conducted using Qiagen's Ingenuity Pathway Analysis software (IPA, QIAGEN Redwood City, www.qiagen.com/ingenuity) using the “Canonical Pathways” and “Diseases & Functions” options. Pathways and functions that surpassed a p<0.05 threshold were reported as significant.

### Statistical analysis

To examine the impact of HIV-1, ART, and gender on gene expression in KS tumors, we compared transcriptome profiles between EpKS and EnKS patients, between ART-treated and–naïve KS patients, and between male and female KS patients. A nonparametric Mann-Whitney U test was used to assess differences between comparison groups. Correlation of biological parameters between groups was done by non-parametric Spearman correlation analysis. GraphPad Prism 5 (Graphpad Software, San Diego, CA) was used for statistical analyses. All tests were 2-tailed, and P-values <0.05 were considered significant.

### Ethics statement

All biological samples in this study were obtained specifically for the study. Approval to conduct the study and all study protocols were obtained from the review boards of Tanzania National Institute for Medical Research, Ocean Road Cancer Institute, University of Zambia Biomedical Research Ethics Committee, and the University of Nebraska-Lincoln (UNL). All participants provided written informed consent.

## Results

### Characteristics of study participants

To investigate the impact of HIV-1, ART, and gender in KS transcriptome profile, we analyzed transcriptome profiles from a total of 51 skin tissues from 27 Tanzanian and Zambian participants (Table 1). Twenty-four KS patients (6 EnKS and 18 EpKS) contributed paired samples from KS lesion and contralateral/ipsilateral control skin. EpKS subjects were of both genders, and further sub-divided into two groups, ART treated (N = 10) and ART naive (N = 8). Seven of the ten ART-treated patients were viral suppressed while the remaining 3 had either less than a month of ART or experienced ART failure. HIV-1 was readily detected in the plasma of all ART-naïve EpKS patients.

**Table 1:** Characteristics of the study subjects.

	ID	Gender	Age	KS months	Plasma KSHV PCR	Plasma KSHV cps/mL	ART status	ART months	Plasma HIV-1 cps/mL	Lesion type
**Healthy skin donors**	3212	M	43	NA	NR	NR	NA	NA	NA	NA
	3214	M	36	NA	NR	NR	NA	NA	NA	NA
	3215	M	47	NA	NR	NR	NA	NA	NA	NA
	
**Endemic EnKS** **HIV-1^-^**	3111	M	24	7	-	NA	NA	NA	NA	Nodular
	3124	M	24	48	-	NA	NA	NA	NA	Nodular
	3135	M	25	12	-	NA	NA	NA	NA	Nodular
	3136	M	45	12	+	BDL	NA	NA	NA	Nodular
	3139	M	27	14	+	BDL	NA	NA	NA	Plaque
	3140	M	66	12	-		NA	NA	NA	Plaque
	
**Epidemic EpKS** **HIV-1^+^**	3122	M	34	3	+	2.8x10^4^	ART treated	24.3	<100	Plaque
	3128	M	28	0.8	+	5.9x10^3^	ART treated	1	<100	Nodular
	3129	M	32	4	+	7.6x10^2^	ART treated	2	<100	Plaque
	21196	F	35	5	-	NA	ART treated	36.5	6x10^5^	Nodular
	21199	F	26	36	-	NA	ART treated	36.5	<100	Nodular
	21221	F	40	5	+	BDL	ART treated	121.7	1x10^7^	Nodular
	032	F	30	3	-	NA	ART treated	0.7	1,300	Plaque
	3022	M	46	12	+	BDL	ART treated	3	<100	Nodular
	3032	M	37	3	+	BDL	ART treated	60	<100	Plaque
	21083	M	54	24	-	NA	ART treated	24	<100	Patch
	023	F	32	7	-	NA	ART naive	0	1x10^8^	Plaque
	025	M	31	2	-	NA	ART naive	0	2.3x10^4^	Nodular
	035	M	31	1	-	NA	ART naive	0	9.5x10^5^	Nodular
	026	M	39	3	-	NA	ART naive	0	5.5x10^4^	Plaque
	034	M	31	3	-	NA	ART naive	0	3x10^7^	Nodular
	037	F	44	3	-	NA	ART naive	0	1.5x10^4^	Plaque
	038	F	55	1	-	NA	ART naive	0	9x10^3^	Nodular
	21214	F	35	1	-	NA	ART naive	0	1.4x10^5^	Plaque

NA–Not Applicable

NR–Not Recorded

BDL–Below Detection Limit

KS–Kaposi’s sarcoma

ART–Antiretroviral Therapy

Cps–Copies

EnKS–Endemic KS

EpKS–Epidemic KS

The median age for EnKS subjects was surprisingly low at 26 years, in comparison to previous reports [41,59]; however, it was not significantly different from that of the regionally comparable EpKS patients at 34 years (P = 0.2). It was notable that EnKS patients self-reported significantly longer duration of KS than EpKS patients (P = 0.006) (S1 Fig) which likely accounts for their advanced disease staging. However, KS duration was not associated with significant transcriptome changes in KS. The majority of KS patients, both EnKS and EpKS, had multiple nodular cutaneous lesions on the extremities with features consistent with T1 staging by the AIDS Clinical Trial Group (ATCG) [60]. Importantly, comparison of transcriptome signatures (lesion/control) between patients with plaque and nodular KS morphotypes showed no significant differences after correction for multiple testing. Three healthy male individuals provided normal skin tissues for transcriptomic comparisons to uninvolved control skin (Table 1). Analysis of HLA allele in lesions and control tissues demonstrated that all paired samples were matched (Table 2). Additionally, since HLA class I molecules can be grouped into supertypes that cluster sets of molecules that bind largely overlapping peptide repertoires [61], we grouped HLA alleles from this cohort according to HLA class I supertypes. Unlike other reports from SSA [62–64], A*02 (13/24, 54%) and B*07 (17/24, 71%) were the major supertypes observed in KS patients (S1 Table). Importantly, country of origin of the participants had no significant impact on studied parameters.

**Table 2:** HLA types and super-types for paired lesion and control.

	Sample ID	A1	A2	B1	B2	C1	C2
Healthy skin donors	3212N	A*30:02	A*68:02	B*42:01	B*15:10	C*07:04	C*17:01
	3214N	A*30:02	A*24:02	B*50:01	B*53:01	C*06:02	C*04:01
	3215N	A*30:04	A*74:01	B*15:03	B*58:02	C*06:02	C*02:10
Endemic EnKS HIV-1^-^	3111C_3111L	A*43:01	A*68:02	B*07:02	B*58:02	C*06:02	C*07:02
	3124C_3124L	A*30:02	A*36:01	B*57:03	B*53:01	C*18:01	C*04:04
	3135C_3135L	A*30:02	A*29:02	B*45:01	B*14:03	C*06:02	C*08:02
	3136C_3136L	A*01:01	A*68:02	B*48:01	B*07:02	C*18:01	C*07:02
	3139C_3139L	A*02:01	A*23:01	B*51:01	B*15:17	C*16:01	C*05:01
	3140C_3140L	A*02:01	A*29:10	B*42:01	B*58:02	C*17:01	C*06:02
Epidemic EpKS HIV-1^+^	3122C_3122L	A*33:03	A*68:02	B*45:01	B*53:01	C*04:01	C*06:02
	3128C_3128L	A*02:01	A*29:02	B*13:02	B*15:03	C*02:10	C*06:02
	3129C_3129L	A*23:01	A*23:01	B*58:01	B*44:03	C*07:01	C*04:01
	21196C_21196L	A*30:02	A*68:01	B*53:08	B*58:02	C*04:01	C*06:02
	21199C_21199L	A*02:02	A*23:01	B*08:01	B*58:01	C*07:01	C*06:02
	21221C_21221L	A*23:01	A*23:01	B*15:03	B*35:01	C*04:01	C*02:10
	032C_032L	A*23:01	A*68:02	B*81:01	B*44:03	C*04:14	C*06:06
	3022C_3022L	A*02:01	A*23:01	B*49:01	B*53:01	C*07:01	C*06:02
	3032C_3032L	A*01:01	A*23:01	B*81:01	B*08:01	C*18:01	C*03:04
	21083C_21083L	A*02:01	A*68:02	B*45:01	B*53:01	C*16:01	C*04:01
	023C_023L	A*33:01	A*66:01	B*58:06	B*53:01	C*04:01	C*06:02
	025C_025L	A*23:01	A*66:01	B*42:01	B*58:02	C*17:01	C*06:02
	035C_035L	A*24:02	A*30:01	B*42:01	B*44:03	C*17:01	C*04:01
	026C_026L	A*03:01	A*30:02	B*45:01	B*58:02	C*16:01	C*06:02
	034C_034L	A*30:02	A*68:02	B*07:02	B*18:01	C*07:04	C*07:02
	038C_038L	A*02:01	A*26:01	B*07:02	B*53:01	C*04:01	C*07:02
	037C_037L	A*34:02	A*74:01	B*44:03	B*58:02	C*04:01	C*06:02
	21214C_21214L	A*02:01	A*30:02	B*14:02	B*58:02	C*06:02	C*08:02

L- Lesion

C–Control

N–Normal/Healthy

EnKS–Endemic KS

EpKS–Epidemic KS

### RNA-seq data quality and alignment statistics

Total RNA from KS lesions, contralateral/ipsilateral control skin and normal skin from healthy donors were sequenced at an average depth of 22.5 million reads (11 to 34 million reads). Overall, the read alignment to human genome ranged between 62% to 83% with approximately 50% of the reads aligning to human exons (32% to 45%) (S2 Table). Unsurprisingly, more reads aligned to the KSHV transcriptome in the KS lesions compared to control and normal skin, ranging from 718 to 31,037 KSHV reads per sample. However, elevated KSHV reads were found in 3 of the control skin samples 3139, 037 and 3122. KSHV reads in these control samples were up to 20% of reads in corresponding lesions (3139–8.7%, 037–19.9% and 3122–20.1% of the lesion) (S2 Table). Due to KS involvement on the contralateral surface, and an intent to sample from a similarly hypoxic region as the KS lesions, these controls samples were collected from grossly uninvolved ipsilateral skin (same side as the site of biopsy). It is possible that elevated KSHV reads in these samples represent a proximity effect or even early KS lesions that were cutaneously inapparent at the time of biopsy. In the other control samples and in the tissues from healthy controls, KSHV reads were less than 2% of the numbers in corresponding lesions, if detected at all. This low-level detection of KSHV reads in control skin tissue could derive from KSHV infected circulating peripheral mononuclear cells (PBMC) moving through tissue vasculature. Interestingly, despite inclusion of HIV-1 infected KS patients previously unexposed to ART, HIV-1 reads were virtually undetectable in the tissues of all EpKS patients (ranging from 0 to 12 reads) (S2 Table) suggesting the lack of a direct role for HIV-1 gene products in the maintenance of KS tumors.

### Similar transcriptome profiles from normal skin of healthy donor and KS patients

**Fig 2:**
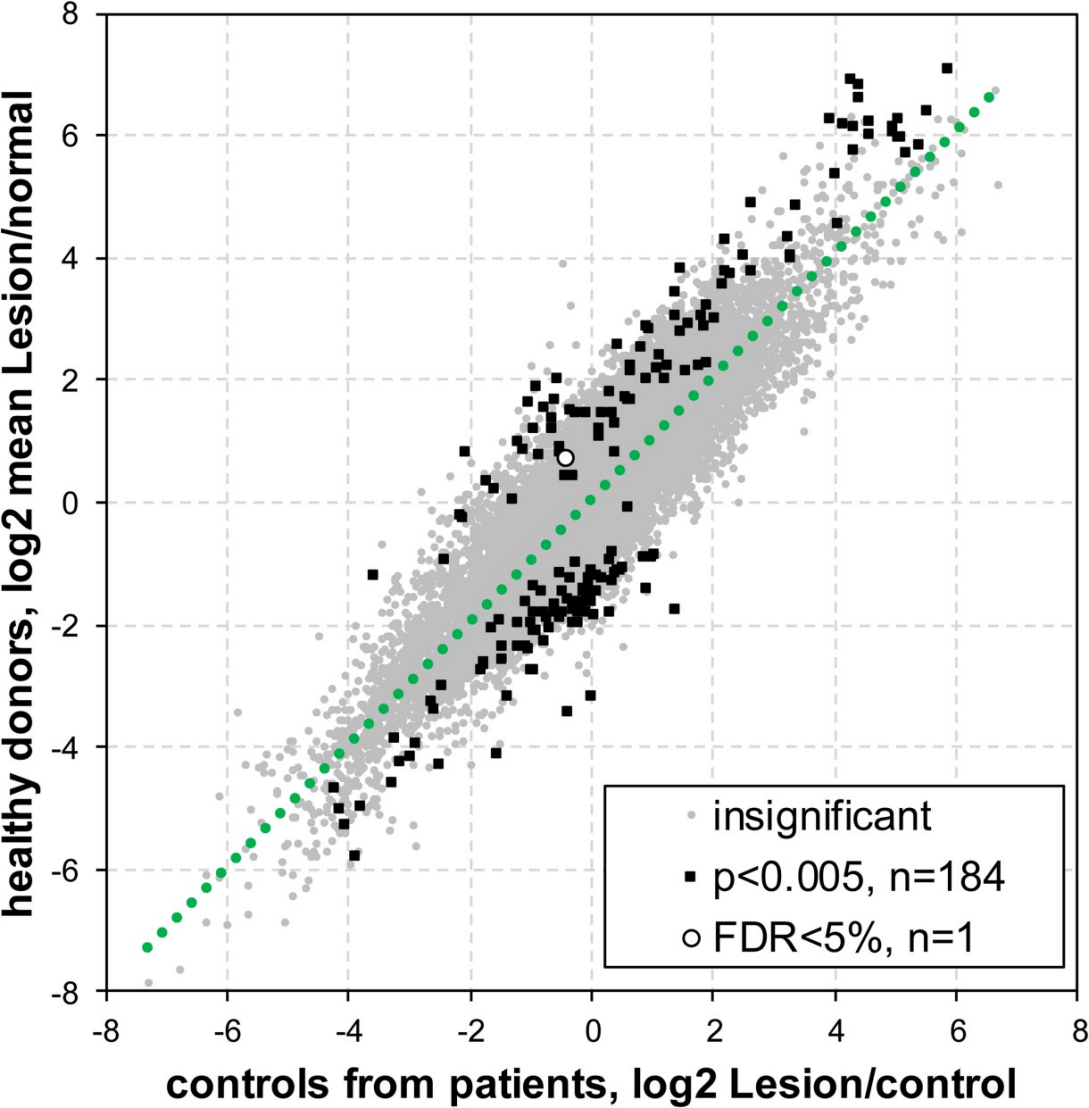
Comparison of cellular gene expression between healthy donors’ skin and control skin from KS patients in a context of differences versus KS lesion. (A) Scatter plot of Lesion/control (mean lesion/mean control from the same patient) and Lesion/normal (mean lesion of all the patients/mean healthy skin samples) expression log_2_-ratios. Regression line is shown as a green dotted line. Gray dots indicate genes whose expression was not significantly different (p>0.005) between healthy skin from donors and control skin from KS patients, squares show genes whose expression was significantly different between healthy skin from donors and control skin from KS patients at P<0.005 (N = 184) with red indicating up- and blue–down-regulation in control as compared to normal skin. Circle marks gene with expression significantly different between healthy skin from donors and control skin from KS patients at FDR<5% (N = 1). FDR–False discovery rate.

To address whether the gene expression patterns in uninvolved control skin of KS patients were reflective of truly normal cutaneous expression since the disease may have a systemic effects to alter cellular gene expression, we compared the transcriptome profiles of uninvolved control KS patient skin to that of normal skin from healthy donors. No major differences in gene expression profiles were observed between health donor skin and control skin from KS patients (Fig 2). Only 184 genes showed significant differential expression even with nominal unadjusted P<0.005. Importantly, analysis of sample variability within and across different samples revealed no obvious pattern indicating high variability between samples (S2 Fig). Due to possible random variability in the data or patients, we detected a single significantly dysregulated gene between uninvolved control and healthy donor skin after correction for multiple testing at FDR <5%. This proved to be the immunoglobulin heavy chain variable segment 7–27 (IGHV7-27) pseudogene, which was upregulated 2.2-fold in normal skin. Overall, the clear conclusion is that the gene expression pattern of the control skin from KS patients reflects those from healthy donors’ skins, suggesting the KSHV dysregulation of gene expression is localized within the KS lesions, and that non-disease skin from the KS patients is an appropriate control for transcriptome changes in KS lesions. This finding underpins and validates the findings from our previous study [29].

### HIV-1 indirectly promotes KS development/maintenance

**Fig 3:**
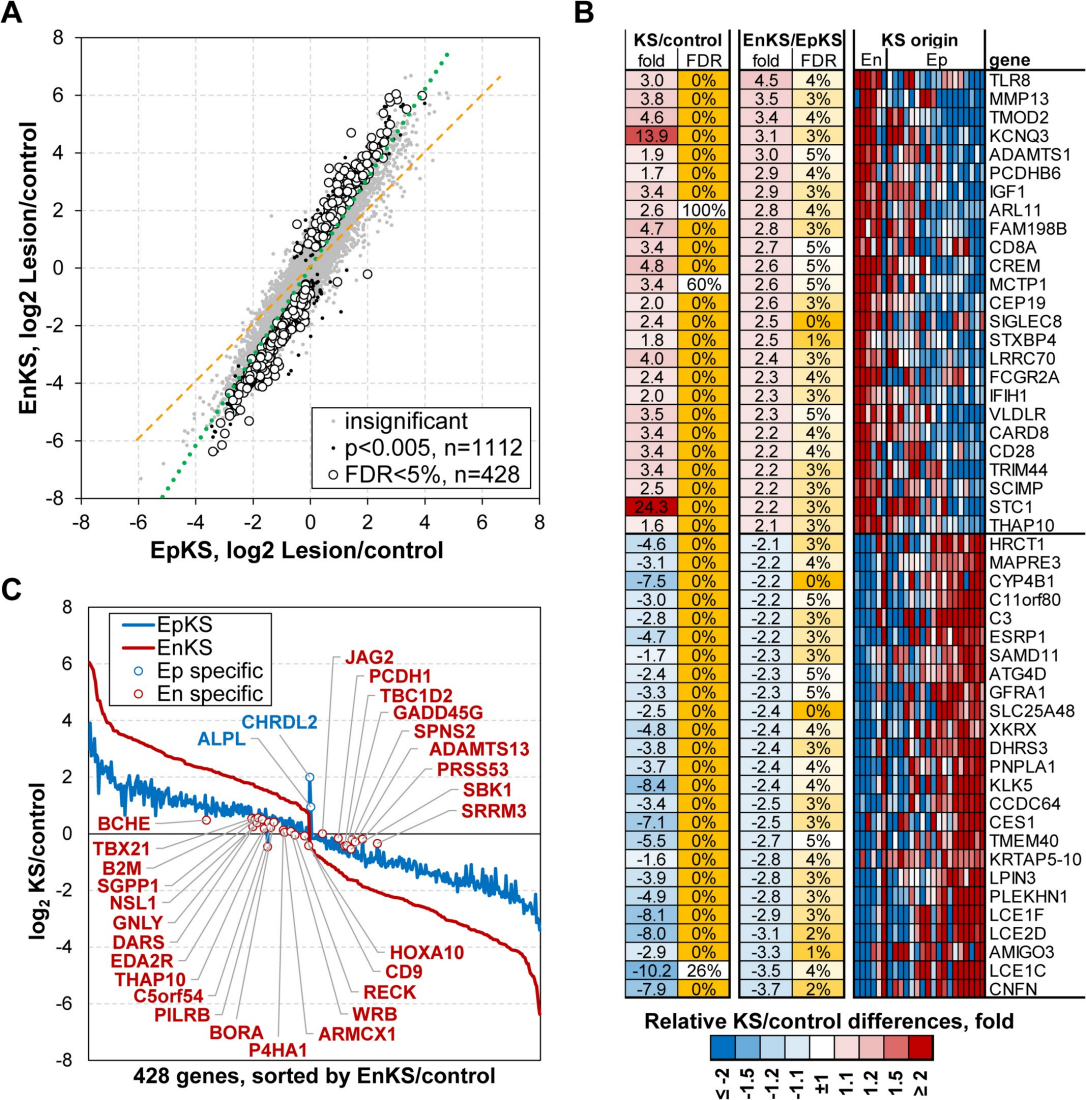
Comparison of transcriptome profiles (KS lesion vs control) between EpKS and EnKS patients. (A) A scatter plot comparing transcriptome profiles between EpKS and EnKS patients. Green dotted line denotes actual regression line; orange dotted line denotes expected relationship without any difference between groups. Grey dots denote genes whose expression was not significantly different between EpKS and EnKS patients. Black dots denote genes whose expression was nominally significantly different between EpKS and EnKS patients at P = 0.005 (N = 1112). Filled circles denote genes whose expression was significantly different between EpKS and EnKS patients at FDR<5% (N = 428). FDR–False discovery rate. (B) Heatmap of top coding genes that are significantly different in KS lesion vs controls and significantly more dysregulated in EnKS than EpKS patients. (C) Comparison of KS lesion vs control differences for 428 genes that are significantly different between EnKS and EpKS indicates that majority of genes are not uniquely dysregulated in endemic KS, but that dysregulation is more extreme in EnKS compared to EpKS. White filled circles highlight 29 genes that had at least 1.5-fold KS lesion/control fold change in one group, but less than 50% of the fold in another group. EnKS–Endemic Kaposi’s sarcoma, EpKS–Epidemic Kaposi’s sarcoma.

HIV-1 infection is epidemiologically associated with KS development, such that up to 50% of untreated HIV-1 co-infected individuals develop EpKS as an AIDS-associated malignancy [31–34]. However, the mechanisms by which HIV-1 infection influences KS development are not completely understood. To investigate whether there exists a direct intra-tumor role for HIV-1 co-infection in KS, we compared transcriptomic differentials between HIV-1 co-infected (EpKS) and HIV-1 uninfected (EnKS) patients. While there was a very strong overall concordance in gene dysregulation between EpKS and EnKS patients (Spearman r = 0.83, p<10^−10^) indicating overall concordance in gene dysregulation. We identified 428 genes that have differences in the magnitude of the differential expression between tumor and uninvolved control tissue between EpKS and EnKS (FDR<5%, Fig 3A). Most of these genes were not uniquely dysregulated in EpKS or EnKS, but rather the magnitude or extent of dysregulation was more pronounced (average of 2.6-fold) in EnKS (Fig 3B and 3C). There were only 29 genes that underwent at least 1.5-fold differential expression in one of the KS types, but not in the other. However, most of those genes (27 genes) were specific to EnKS (Fig 3B). Expression of differential genes between EnKS and EpKS was variable as shown by an MA plot (S3 Fig).

Enrichment analysis of the significant differences between EpKS and EnKS using the Ingenuity Pathway Analysis (IPA) suggested enhanced dysregulation of functions like tumorigenesis and inflammatory/immune responses in EnKS compared to EpKS. For example, microtubule associated protein RP/EB family member 3 (MAPRE3) a known oncogene is differentially expressed in EpKS compared to EnKS (Fig 3B) [65,66]. Epithelial splicing regulatory protein-1 (ESRP1) that signals through oncogenic MAPK signaling pathway is also differentially dysregulated in EpKS compared to EnKS patients. ESRP1 has been shown to support anchorage-independent growth of colorectal cancer cells and promote metastasis formation *in vitro* [67,68]. It also supports mesenchymal to epithelial transition in ovarian cancer cells [69]. Genes related to immune responses like CD28 and CD8A, involved in activation of CD8+ T-cells were also differentially expressed in EpKS compared to EnKS patients. Overall, the KS transcriptome in EpKS and EnKS were surprisingly similar except for a small number of genes where the magnitude of gene dysregulation was more pronounced in EnKS compared to EpKS. This suggests that despite the lack of detection of HIV-1 gene expression in EpKS tumors, KSHV requires a greater extent of cellular reprogramming of tumorigenesis- or inflammation/immune response-associated gene to induce cancer on its own than when in the presence of HIV-1 co-infection.

### Similar transcriptome profiles in KS patients regardless of gender or ART-use

**Fig 4:**
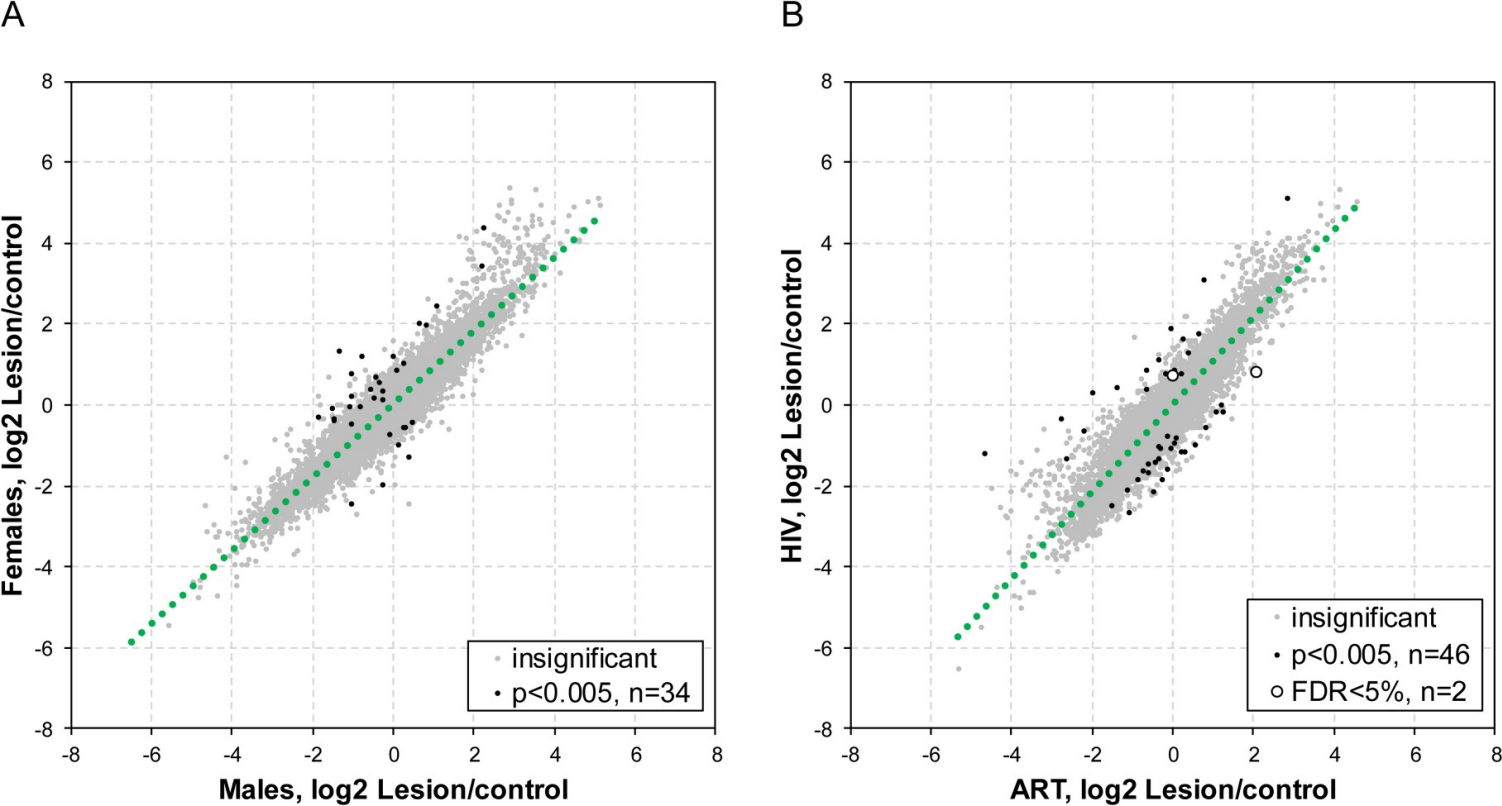
Comparison of transcriptome profile in KS patients by gender and by ART-use. (A) A scatter plot comparing transcriptome profiles between male and female EpKS patients. Green dotted line denotes regression line. Grey dots denote genes whose expression was not significantly different between male and female EpKS patients, black dots denote genes whose expression was significantly different between male and female EpKS patients at P = 0.005 (N = 34). (B) A scatter plot comparing transcriptome profiles between ART treated and ART naïve KS patients. Green dotted line denotes regression line. Gray dots denote genes whose expression was not significantly different between ART treated and ART naïve KS patients, black dots denote genes whose expression was significantly different between ART treated and ART naïve KS patients at P = 0.005 (N = 46) and unfilled circles denote genes whose expression was significantly different between ART treated and ART naïve KS patients at FDR<5% (N = 2). FDR–False discovery rate. EnKS–Endemic Kaposi’s sarcoma, EpKS–Epidemic Kaposi’s sarcoma.

To explore the potential transcriptomic basis for gender in KS development, we compared transcriptomic differentials between male and female EpKS patients. Most of the dysregulated cellular genes were similar between male and female EpKS patients. Despite marginal differential expression in a few genes (n = 34) between male and female, none of these genes were significant at FDR<5% (Fig 4A). Comparison of absolute normalized expression between male and female showed genes with conserved differences between classes in controls and lesions demonstrating strong non-KS specific effect of patient cohorts on baseline expression levels (S4A Fig). However, our analysis only included EpKS patients where gender differences are known to be less obvious. Since our EnKS cases are all males, further analysis of gender in EnKS patients is clearly warranted to define the role of gender in KS development.

The use of ART to control HIV-1 has been associated with a number of cellular changes [44–47]. Since EpKS patients in our cohort had varying ART duration and some with detectable plasma HIV-1 viral load despite ART treatment, we tested the effect of ART duration and HIV-1 viremia on EpKS transcriptome profiles. Duration of ART was not associated with KS lesion transcriptome profile as highlighted (S5A Fig and S5B Fig). Similarly, HIV-1 viremia had no significant effect on KS transcriptome profiles (S5C Fig). Only a few genes (33 genes, <1%) showed significant changes in gene expression profiles between HIV-1 viremic and aviremic EpKS patients, and none passed significance thresholds after correction for multiple testing (S5D Fig). To determine whether ART use was responsible for a subset of the cellular metabolic changes detected in KS lesions, we compared transcriptomes between ART treated and ART naïve EpKS patients. No major differences in gene expression were observed between ART-treated and -naïve EpKS patients (Fig 4B). Only 46 genes had nominal P<0.005 with mild changes between ART naïve and experienced patients but only 2 of these genes were significant at FDR<5% (Fig 4B). Comparison of absolute normalized expression between ART-treated and -naïve EpKS patients showed genes with conserved differences between classes in controls and lesions demonstrating strong non-KS specific effect of patient cohorts on baseline expression levels (S4B Fig). Taken together, we identified no significant effect on overall gene expression in KS tissues versus uninvolved control skin due to ART use in EpKS patients.

### Diversity of KSHV gene expression in KS lesions

KSHV predominantly adopts a latent gene expression pattern in infected cells where a few viral genes including latency-associate nuclear antigen, LANA (ORF73), viral cyclin D homolog, vCYC (ORF72), Kaposin (K12) and viral Fas-associated protein with death domain-like interleukin 1β converting enzyme/caspase-8- inhibitory protein, vFLIP (ORF71), are expressed to maintain the viral episome [70,71]. Apart from maintaining the viral episome, these KSHV genes have been associated with malignant transformation [72–76]. LANA has been suggested to suppress p53 whereas vCyclin degrades Rb, vFLIP impairs recruitment and activation of caspase 8 to block apoptosis and Kaposin plays a role in transformation and cytokines production [72]. At low frequency, spontaneous or induced lytic reactivation of KSHV occurs leading to lytic gene expression and production of early lytic gene products important for pathogenesis or results in production of viral progeny. The replication transactivator (RTA) acts as a switch from KSHV latent to lytic life cycle through interactions with various cellular transcription factors leading to lytic KSHV replication [77,78]. Similarly, some KSHV lytic genes like K1, K15, vIL6 and vGPCR have also been implicated in KS formation [72–76].

**Fig 5:**
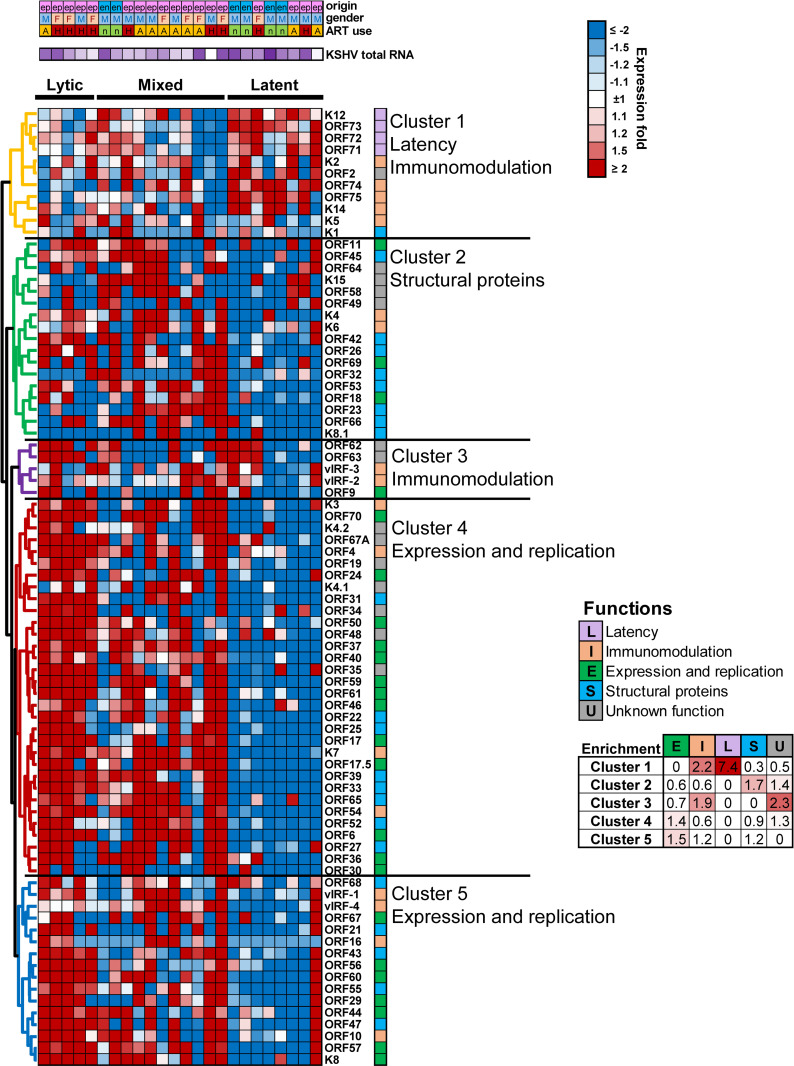
Hierarchical clustering of KSHV genes and samples. ART–Antiretroviral therapy, KSHV–Kaposi’s sarcoma (KS)-associated herpesvirus, en–endemic KS, ep–epidemic KS, M–Male, F–Female, A–ART treated, H–ART naïve, n–HIV-1 negative.

To investigate the KSHV gene expression profile in KS lesions, expression of KSHV genes from each lesion were normalized to the respective total KSHV RNA reads and subjected to unsupervised hierarchical clustering. Consistent with previous studies, three gene expression patterns were observed in both EpKS and EnKS tumors (Latent, lytic and intermediate/mixed) (Fig 5) [79,80]. Lesions exhibiting the latent pattern had low-level KSHV gene expression throughout the genome outside of the latency locus. A subset of KS lesions had a mixture of both latent and lytic gene expression patterns (Fig 5). However, in subjects with the lytic expression pattern, the viral gene expression did not correlate with detection of KSHV plasma viremia (Table 1). KSHV virion DNA was detectable in 4 out of 10 patients with mixed latent and lytic, 1 in 6 patients with lytic and 2 out of 8 patients with latent viral gene expression profiles. While EpKS patients were distributed in all 3 gene expression patterns, EnKS patients displayed mainly latent and mixed expression profiles. Similarly, gene expression patterns were not segregated according to ART-use or gender (Fig 5). Overall, KSHV gene expression patterns in KS lesions were variable in both EnKS and EpKS patients.

Furthermore, irrespective of HIV-1 infection status, five clusters of KSHV genes were observed in KS lesions according to known gene functions (Fig 5). Although most clusters separated according to gene functions, overlap of functions was also observed. Enrichment analysis of genes in each cluster suggested that Cluster-I included genes involved in latency and immunomodulation, Cluster-II was primarily genes for structural proteins, and Cluster-III included genes involved in host immunomodulation. Both Clusters IV and V were enriched with genes involved in viral expression and replication. KSHV gene clusters contained a mixture of EpKS and EnKS patients, who were ART-experienced or -naïve and of both genders. Overall, the expression patterns and gene clusters did not segregate according to HIV-1 co-infection status (EnKS versus EpKS), ART-use or gender (Fig 5).

### KSHV induced cellular changes lead to increased cell survival in KS lesions

**Fig 6:**
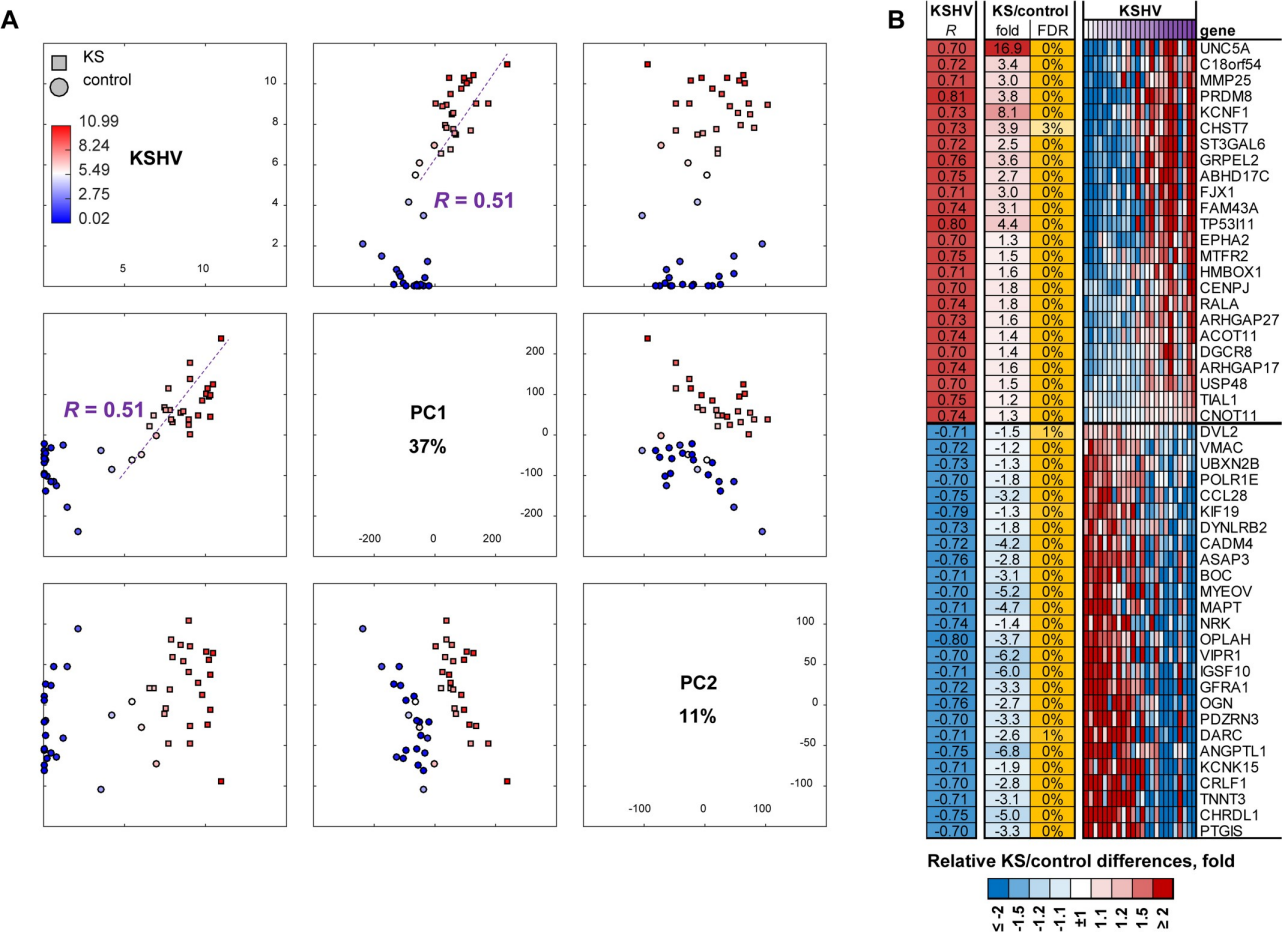
Correlation between KSHV RNA load/reads and cellular gene expression. (A) Principal component analysis (PCA) showing relationship between first and second principal components (PC1, PC2) and KSHV RNA load/RNA in KS lesion vs control. (B) Heatmap for top 50 genes correlated with KSHV RNA load/read that were also up- or down-regulated in KS lesions vs controls. KSHV–Kaposi’s sarcoma (KS)-associated herpesvirus.

Because KSHV is a necessary, but insufficient, agent in KS development, we investigated relationships between KSHV RNA load/reads and cellular gene expression in KS lesions and controls. Principle component analysis (PCA) demonstrated the anticipated separation of lesion and control samples within the first two principle components (PC1 explaining 37% and PC2–11% of all data variability) (Fig 6A). Increase in KSHV RNA load/reads in the KS lesions correlated with dysregulation of cellular gene expression (Pearson r = 0.51, P = 0.01), indicating that cellular gene expression is greatly affected by KSHV in both EpKS and EnKS lesions (Fig 6A). Direct analysis of genes that were differentially regulated in lesion tissue versus uninvolved control skin for correlation with KSHV RNA load, revealed 866 genes that were significantly dysregulated in KS (FDR<5%) and significantly correlated with KSHV RNA load/reads (FDR<10%) (Fig 6B). The top 50 differentially regulated cellular genes correlating with KSHV tissue load are shown in Fig 6B. Enrichment analysis of these genes by IPA showed functions associated with decreased apoptosis and increased cellular proliferation, angiogenesis, and tumorigenesis in EpKS and EnKS.

### Consistent gene expression pattern underlying KSHV pathogenesis

**Fig 7:**
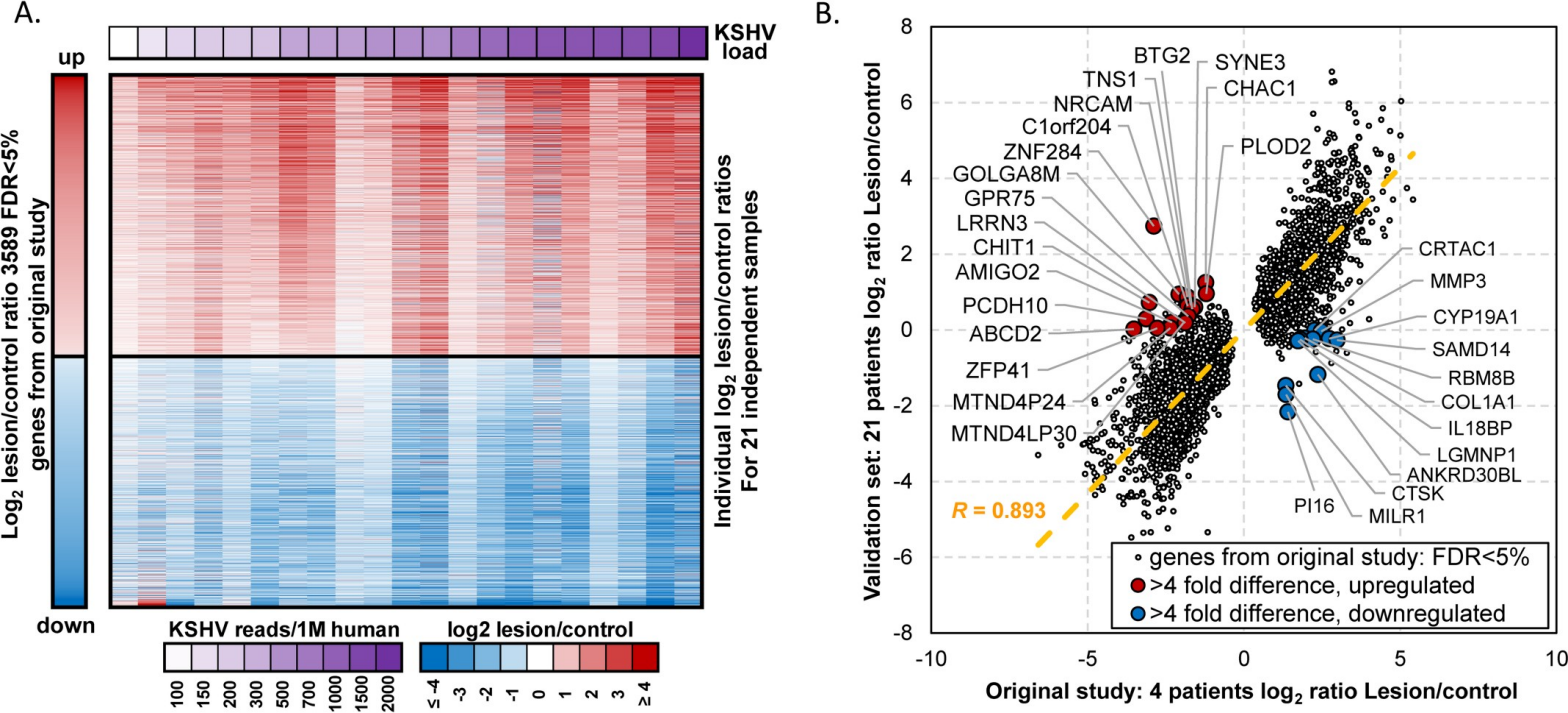
Correlation of cellular gene expression in KS lesion/control between previous and current study. (A) Heatmap of lesion/control difference in expression from 21 new sample pairs for 3589 up- or down-regulated cellular genes reported in the previous study. (B) Correlation of cellular genes differences between KS lesion vs control reported in the previous study versus current study. Top changes for 30 up- and down-regulated genes (>4-fold change) unique to the current study are highlighted in red and blue, respectively. Correlation line is shown by dotted orange line. FDR–False discovery rate. KSHV–Kaposi’s sarcoma (KS)-associated herpesvirus.

**Fig 8:**
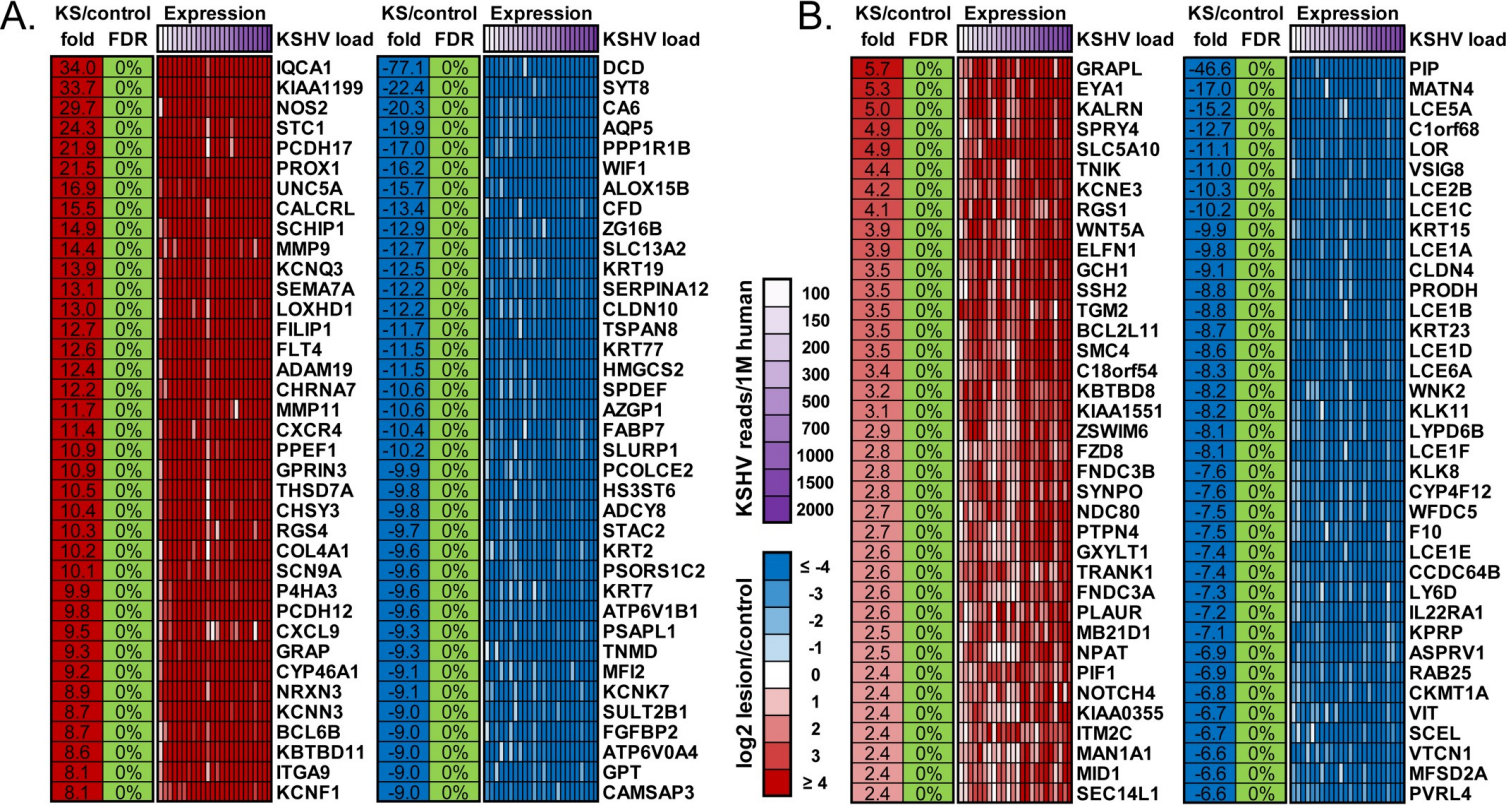
Expression heatmap for cellular genes. (A) Overlapped with previous study and (B) uniquely found in the current study. FDR–False discovery rate. KSHV–Kaposi’s sarcoma (KS)-associated herpesvirus.

In addition to investigating the impact of HIV-1, ART, and gender on transcriptome profiles of KS patients, we determined gene expression patterns associated with KSHV pathogenesis. We compared our previous dataset (4 paired EpKS samples) with the additional sample pairs from the current dataset (21 paired samples). Of the 3589 genes reported to exhibit significantly dysregulated expression versus uninvolved control skin in the previous study at FDR<5%, 96.4% showed the same direction of change in the completely independent and much larger cohort of patients. Overall expression patterns were similar (Fig 7A), with only 30 genes demonstrating lack of change of 4-fold or more. Of the 1193 genes reported to be involved in significant functions, pathways and regulators reported in the previous study, 97.2% were also validated to have the same direction of change in the current study. Importantly, the magnitude of change in gene expression in KS lesions versus controls significantly correlated between previous and current study (r = 0.89, p<10^−10^, Fig 7B). While the direction of differential expression remained the same for most genes, the magnitude of change for several genes increased or decreased with the additional samples in the current study. The top 37 up- and down-regulated coding-genes overlapping between the previous and current study are shown in Fig 8A. The additional samples revealed 522 genes that previously did not reach significance in differential expression due to the limited sample size but were found to be significantly up- or down-regulated in the current larger study. The top 37 up- and down-regulated genes unique to the current study (FDR<5%) are highlighted in the heatmap in Fig 8B. Overall, there is over 95% concordance in gene expression profiles between the previous study and the much larger independent set of samples (current study), highlighting high transcriptome similarities of KS lesions.

## Discussion

To our knowledge, this is the first study to analyze the effects of HIV-1 co-infection on the gene expression profiles in KS lesions with appropriate infection, treatment, and uninvolved tissue controls. This was conducted by comparatively analyzing the transcriptome profiles between EpKS and EnKS patients from SSA. The study involved sequencing 51 individual skin tissues to support investigation of multiple comparison groups to better characterize the impact of HIV-1, ART, and gender, on KS tissue gene expression profiles. For example, gene expression in uninvolved control skin from KS patients was demonstrated to be indistinguishable one from another, and from skin from non-diseased healthy subjects. So uninvolved skin in a KS patient is indeed ‘normal’ skin. Overall, gene expression profiles correlated significantly between our current and previous study (Spearman r = 0.89, P<10^−10^) [29]. Here we detected significant dysregulation of 3589 of the genes reported in the previous study at an FDR<5%, and 96.4% of those showed the same direction of change. The high similarity between KS transcriptomes derived from the two independent data-sets suggests that the gene expression patterns underlying KSHV pathogenesis are consistent across the spectrum of subjects possessing attributes that we might previously have predicted to introduce profound variability into the tumor transcriptomes. In both datasets, HIV-1 transcripts were undetectable, or were at levels indistinguishable from uninvolved control skin, in EpKS transcriptomes [29], suggesting that the impact of HIV-1 coinfection on KS gene expression is indirect or is unnecessary once neoplasia has initiated.

Comparison of control-normalized EpKS and EnKS transcriptomes revealed that up- and down-regulated cellular genes in EpKS and EnKS were highly concordant (r = 0.83, p<10^−10^). Our analyses showed that most of the dysregulated genes involved in immune function and tumorigenesis were shared between the EpKS and EnKS datasets. This is consistent with our recent analysis of peripheral immune responses between ART-treated EpKS and EnKS patients, in which HIV-1 co-infection did not differentially impact cytokine and antibody responses [41]. Nevertheless, a small number of differences were revealed. A subset of genes involved in tumorigenesis and inflammation/immune responses were demonstrated to show differentials in the magnitude of dysregulation between EpKS and EnKS. For these genes, the change in expression between tumor and uninvolved control tissue was slightly, but significantly, higher in EnKS than in EpKS. For example, insulin-like growth factor 1 (IGF1), a potent growth factor that regulates glucose transport and glycogen synthesis was 2.9-fold upregulated in EnKS compared EpKS. It is possible that IGF1 participates in Warburg-like metabolism that we have associated with KS tumorigenesis. Additionally, the expression of CD8A and CD28, two T-cell surface glycoproteins essential in CD8 T-cell stimulation and co-stimulation, was also higher in EnKS compared to EpKS. However, through viral encoded K3 and K5, KSHV escapes detection by cytotoxic T-cells and NK cells by down-regulating MHC class I and other T-cell co-stimulatory molecules like CD86 and ICAM [81,82] so the impact of the detected changes is unclear. Similarly, expression of interferon-induced helicase C domain-containing protein 1 (IFIH1) was higher in EnKS compared to EpKS lesions. It is possible that KSHV manipulation of IFIH1, an innate immune sensor of viral nucleic acids associated with type-1 interferon antiviral responses, could be important in immune evasion. Furthermore, a recently identified oncogene, microtubule associated protein RP/EB family member 3 (MAPRE3) was highly expressed in in EnKS compared to EpKS. MAPRE3 maintains cell polarity, spindle function and promotes malignant cell invasion and metastasis [83]. These differentials in the magnitude of gene expression between EnKS and EpKS for a subset of genes, suggest that, despite suppression of HIV-1 plasma viral load, and lack of detectable HIV-1 gene expression in EpKS tumors, the systemic environment has been altered by HIV-1 infection such that less cellular dysregulation is required to induce or maintain KS neoplastic growth than in EnKS. In other words, it takes a greater extent of cellular reprogramming for KSHV to induce cancer on its own than when in the presence of HIV-1 co-infection. This suggests that HIV-1 co-infection increases the likelihood of KS development. This might explain the increased incidence, acceleration, and exacerbation of KSHV pathogenesis and KS in HIV-1 co-infection as observed in HIV-1 and KSHV co-endemic regions of SSA. However, while it is plausible that, the difference in magnitude of gene expression between EnKS and EpKS could result from longer duration of KS in EnKS as shown in S1 Fig, correlation analysis between KS duration and transcriptome changes did not reach statistical significance.

Several studies, including ours, have reported KSHV induced metabolic alterations *in vitro* and *in vivo* [29,42–44,46,47]. We previously detected profound alterations in glucose and lipid metabolic pathways in ART treated EpKS lesions, but we could not rule-out the role of ART in the observed lipid metabolic changes since all patients were ART treated. In the current study, the comparison of ART-treated versus naïve EpKS expression patterns reveals that the metabolic alterations are not a function of ART or even a direct consequence of HIV-1 infection, but rather, are a central aspect of KS tumor metabolism. It is possible that KS induced glycolysis, in the absence of a properly functioning tricarboxylic acid cycle or oxidative phosphorylation pathway, leads to accumulation of lactate and a metabolic microenvironment that promotes KSHV persistence, tumorigenesis and lack of effective immune surveillance.

A gender differential in incidence is obvious in the epidemiology of endemic and classical KS where more males are affected [48–52]. The mechanisms responsible for this bias, which is less apparent in EpKS, are unknown. Despite prior suggestions of a role of androgen receptors from *in vitro studies* [84], transcriptome profiles of male and female EpKS patients were not differential in this cohort. Perhaps, analysis of gender exclusively in EpKS patients limited our potential to identify differentials, since the effect of gender is less pronounced in EpKS patients. Unfortunately, all recruited EnKS cases were male, as expected given the paucity of female EnKS. To explore the potential transcriptomic bases for gender bias in EnKS development, further analysis would need to include both male and female EnKS patients.

Consistent with the highly vascular tumor tissue containing immature blood vessels that is the KS clinical presentation [85,86], there was evidence for up-regulation of vascular growth factors like angiopoietin-2 (ANGPT2) and at least three vascular endothelial growth factor (VEGF) receptors such as neuropilin-2 (NRP2), fms-related tyrosine kinase (FLT4) and kinase insert domain receptor (KDR) and associated members of this angiogenesis signaling pathway. KS may trigger angiogenesis as a function of the robust infection of CD34^+^ endothelial precursors, or it may occur in response to the nutrient needs of the inefficient glycolytic KS tumor metabolism, described above. Similarly, some gene functions were also involved in increased cell survival and tumor formation, consistent with the oncogenic roles of KSHV [87,88].

Analysis of KSHV gene expression patterns in Ugandan KS patients led to categorization of KS lesions into predominantly latent, lytic or intermediate KSHV gene expression groups [30]. Our current analyses of 48 KS lesions versus uninvolved control skin also revealed three higher order expression patterns: a predominantly latent KSHV gene expression pattern was evident in 33.3% of lesions; a varying extent of lytic expression was evident in the remainder ranging from mixed latent/lytic (41.6%) to nearly exclusively lytic (25%). Lytic expression in such samples could be due to spontaneous and variable reactivation from a limited number of KSHV infected cells whereas latent gene expression may be more important for maintenance and propagation of the tumor [29,30]. Despite the lytic gene expression pattern in some KS lesions, it appears that there was only low-level virus production since KSHV viral DNA was below the detection limit in plasma in ~70% of the patients. Importantly, the KSHV gene expression pattern in KS lesions was unaffected by HIV-1 co-infection, ART-use, or gender. Moreover, consistent with other studies [29,30], genes associated with KSHV latency and host immune-modulation were highly expressed in all KS lesions. It is therefore possible that expression of these core KSHV immune modulating genes, like K2, vIRF1, ORF74 and LANA support immune evasion, as well as metabolic dysregulation and tumor propagation.

KSHV neutralizing antibody (nAb) has been shown to lack protective efficacy against natural KSHV infection. Rather, nAb correlates with KS pathogenesis [41,89]. Only HLA class I supertype A*02 T-cell responses against KSHV have been reported. These T-cell responses against KSHV peptides are generally weak and diverse in both KS patients and asymptomatic controls [90–92]. In this study, we investigated HLA supertypes and their potential association with KS. Our findings had little overlap with those from a recent larger cohort study conducted in Cameroon [64]. A*02 and B*07 supertypes were the most common supertypes in our KS patients as compared to B*27 and B*44 in Cameroon. Substantially different ethnic origins of the subjects sampled at distinct sites on the continent could be one of the reasons for the differential HLA-associations. Larger studies, preferably those coupled to immune functional assessments in the peripheral or tumor infiltrating T-cells, are needed to evaluate the role of HLA types or supertypes in KS development.

Our study expands significantly upon the previous findings derived from comparisons of 4 EpKS and contralateral uninvolved control skin samples solely from ART treated patients [29]. As reported by Kist *et al* [93], one control skin sample in that analysis was even sequenced twice. Here, we show a much more comprehensive analysis of a substantially more encompassing set of KS patients and controls that not only provides the new insights summarized above but has confirmed the overall conclusions of our previous study. Overall, as in all whole tissue RNA-seq experiments, our analysis of KS lesions is partially confounded by the cellular complexity of the tumors; whereas, analysis of separate cell lineages or single cell RNA-seq require *a priori* partitioning decisions that could limit the capacity to detect globally important transcriptome changes in KS. Also, as a limitation of RNA-seq technique and its processing algorithms, for example, it cannot accurately distinguish reads coming from ORF74 versus K14 (vOX-2), as the NCBI KSHV version NC_009333.1 has ORF74 gene (https://www.ncbi.nlm.nih.gov/gene/4961460) sequence being completely within longer sequence of K14 (https://www.ncbi.nlm.nih.gov/gene/4961465). However, the current analysis, in addition to validating our previous findings on altered glucose and lipid metabolism, has addressed the role of HIV-1 and whether a gene expression bias exists based on gender in EpKS patients. Relationships between HLA and risk of KS are still unclear and complicated by lack of functional immune response/repertoire data for haplotypes other than HLA-A*0201. Sequencing 51 individual biopsies from 27 individuals makes this one of the very few if not the only KS study with a large transcriptome dataset. However, distribution of the samples into multiple comparison groups has led to lower sample sizes in some of the groups. This could have limited our ability to detect transcriptome differentials between some groups. Studies with larger sample size particularly with EnKS are warranted to fully characterize KS transcriptome. In conclusion, transcriptome profiling reveals only minor differences between EpKS and EnKS, and no detectable contribution from gender or ART use. The trend toward higher magnitude of gene dysregulation in EnKS coupled with the absence of HIV-1 transcripts in EpKS may suggest an indirect or systemic effect of HIV-1 to promote KS tumorigenesis. Alternatively, KSHV requires greater extent of cellular reprogramming to induce cancer on its own than when in the presence of HIV-1 co-infection.

## Supporting information

S1 Fig Self-reported KS duration in months between EnKS and EpKS patients. EnKS–Endemic Kaposi’s sarcoma, EpKS–Epidemic Kaposi’s sarcoma.
(TIF)

S2 Fig Expression scatter plots demonstrating variation within and across different sample types. N—normal/healthy, L–Lesion and C–Control.
(TIF)

S3 Fig MA plot for significance of expression differences between EnKS and EpKS of lesion/control ratios. (TIF)

S4 Fig Comparison of absolute normalized expression in controls and lesions. (A) Comparison of absolute normalized expression in controls and lesions between male and female. (B) Comparison of absolute normalized expression in controls and lesions between ART-treated and HIV (ART-naive) patients.
(TIF)

S5 Fig Comparison of transcriptome profile in EpKS patients by ART duration and by HIV-1 viremia. (A) A scatter plot comparing transcriptome profiles between EpKS patients treated with ART for more one week (N = 10) and those who were ART naïve or treated for less than a week (N = 8). Green dotted line denotes regression line. Grey dots denote genes whose expression was not significantly different between EpKS patients treated with Art for >1 week and those who are ART naïve of treated for <1 week. Black dots denote genes whose expression was significantly different between EpKS patients treated with Art for >1 week and those who are ART naïve of treated for <1 week at P = 0.005 (N = 23) and unfilled circles denote genes whose expression was significantly different between EpKS patients treated with Art for >1 week and those who are ART naïve of treated for <1 week at FDR<5% (N = 0). (B) A scatter plot comparing transcriptome profiles between EpKS patients treated with ART for more one month (N = 9) and those who were ART naïve or treated for less than a month (N = 9). Green dotted line denotes regression line. Grey dots denote genes whose expression was not significantly different between EpKS patients treated with Art for >1 month and those who are ART naïve of treated for <1 month. Black dots denote genes whose expression was significantly different between EpKS patients treated with Art for >1 month and those who are ART naïve of treated for <1 month at P = 0.005 (N = 46) and unfilled circles denote genes whose expression was significantly different between EpKS patients treated with Art for >1 month and those who are ART naïve of treated for <1 month at FDR<5% (N = 2). (C) A scatter plot comparing transcriptome profiles between EpKS patients with detectable plasma HIV-1 viral load (viremic) (N = 11) and undetectable plasma HIV-1 viral load (aviremic) (N = 7). Green dotted line denotes regression line. Grey dots denote genes whose expression was not significantly different between HIV-1 viremic and aviremic EpKS patients. Black dots denote genes whose expression was significantly different between HIV-1 viremic and aviremic EpKS patients P = 0.005 (N = 33) and unfilled circles denote genes whose expression was significantly different between HIV-1 viremic and aviremic EpKS patients at FDR<5% (N = 0). (D) Principal component analysis (PCA) showing relationship of samples on first and second principal components based on transcriptome profile of 33 genes affected by ART in HIV-1 suppressed patients (green circles) vs patients with HIV-1 viremia (red squares) on at nominal p<0.005 in EpKS lesions. ART treated patients with detectable HIV-1 (white triangles) demonstrate similarity to ART naïve, HIV-1 viremic EpKS patients indicating that these genes represent signature of HIV-1 viremia. EnKS–Endemic Kaposi’s sarcoma, EpKS–Epidemic Kaposi’s sarcoma.
(TIF)

S1 Table Frequency distribution of HLA supertypes in healthy donors, EnKS and EpKS patients. EnKS–Endemic Kaposi’s sarcoma, EpKS–Epidemic Kaposi’s sarcoma.
(DOCX)

S2 Table RNA-seq sample quality and alignment statistics. L–Lesion, C–Control, N–Normal/Healthy, KSHV–Kaposi’s sarcoma-associated herpesvirus, HIV-1 –Human Immunodeficiency virus type 1
(DOCX)
