# Supplementary figures and images for: Comparative transcriptome analysis of endemic and epidemic Kaposi’s sarcoma (KS) lesions and the secondary role of HIV-1 in KS pathogenesis
Source: PLoS Pathog. 2020 Jul 24;16(7):e1008681. doi: 10.1371/journal.ppat.1008681 (PMC7406108; doi:10.1371/journal.ppat.1008681)

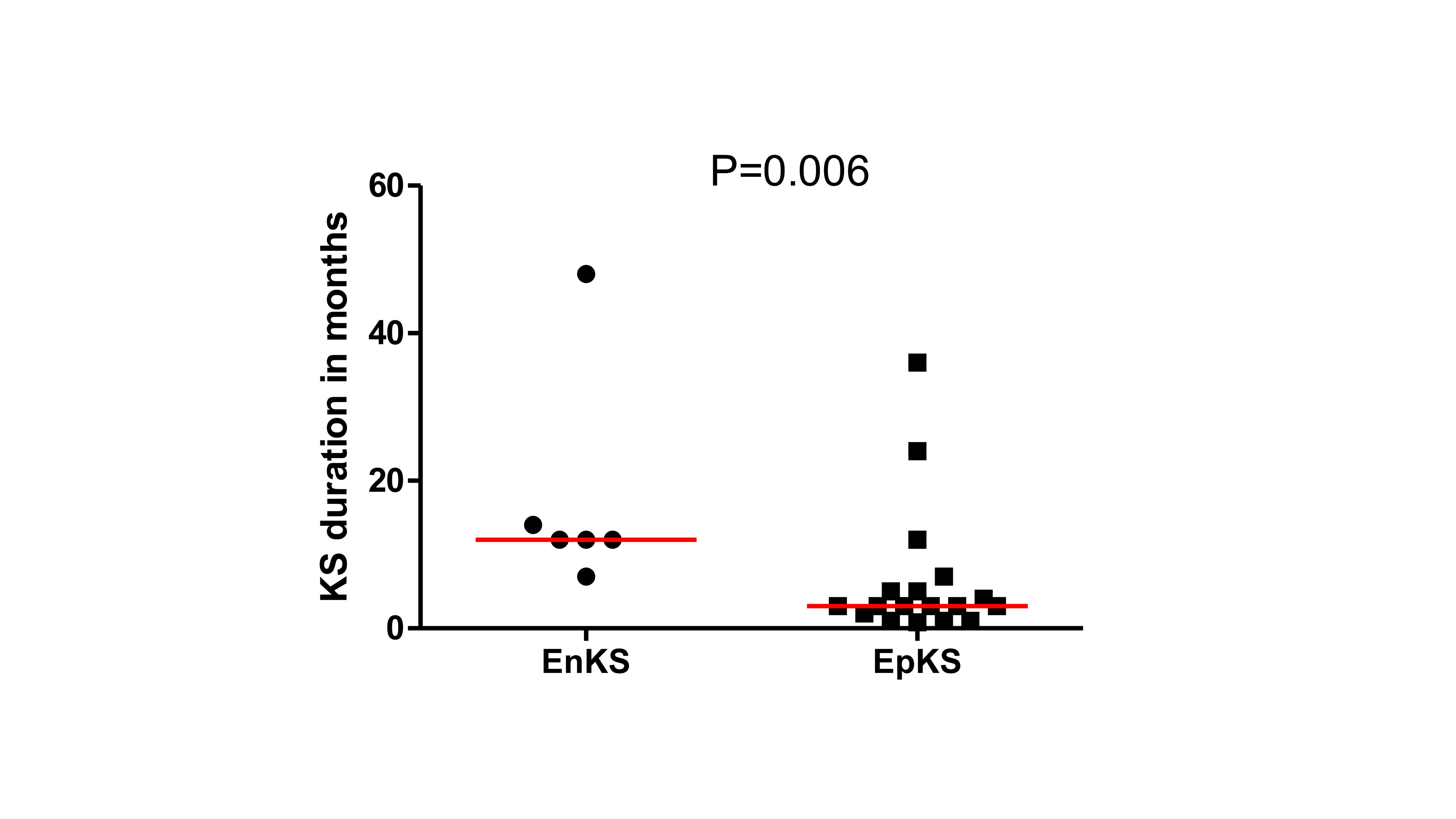

Supplement: S1 Fig — EnKS–Endemic Kaposi’s sarcoma, EpKS–Epidemic Kaposi’s sarcoma. (TIF) [file ppat.1008681.s001.tif]

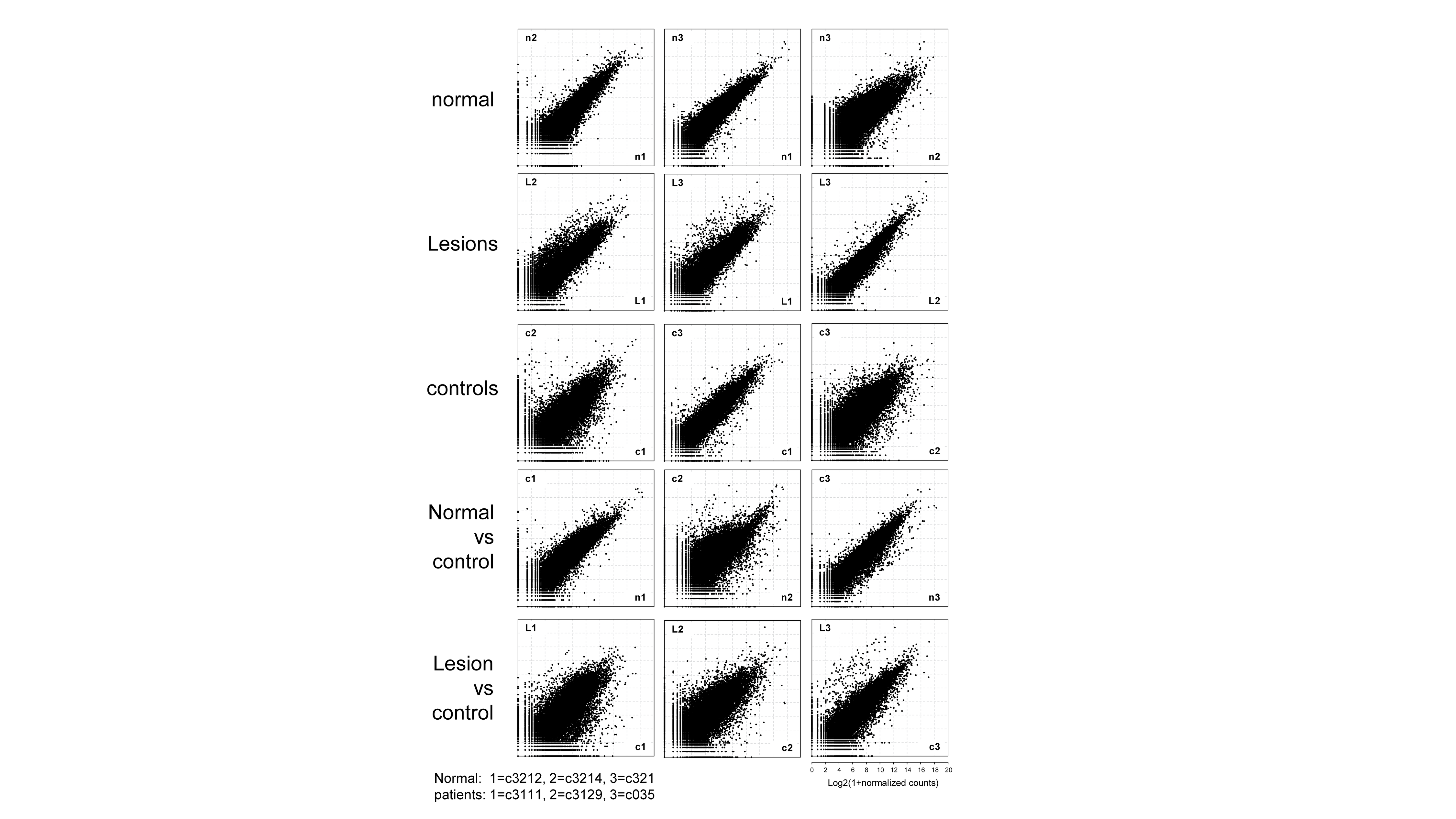

Supplement: S2 Fig — N—normal/healthy, L–Lesion and C–Control. (TIF) [file ppat.1008681.s002.tif]

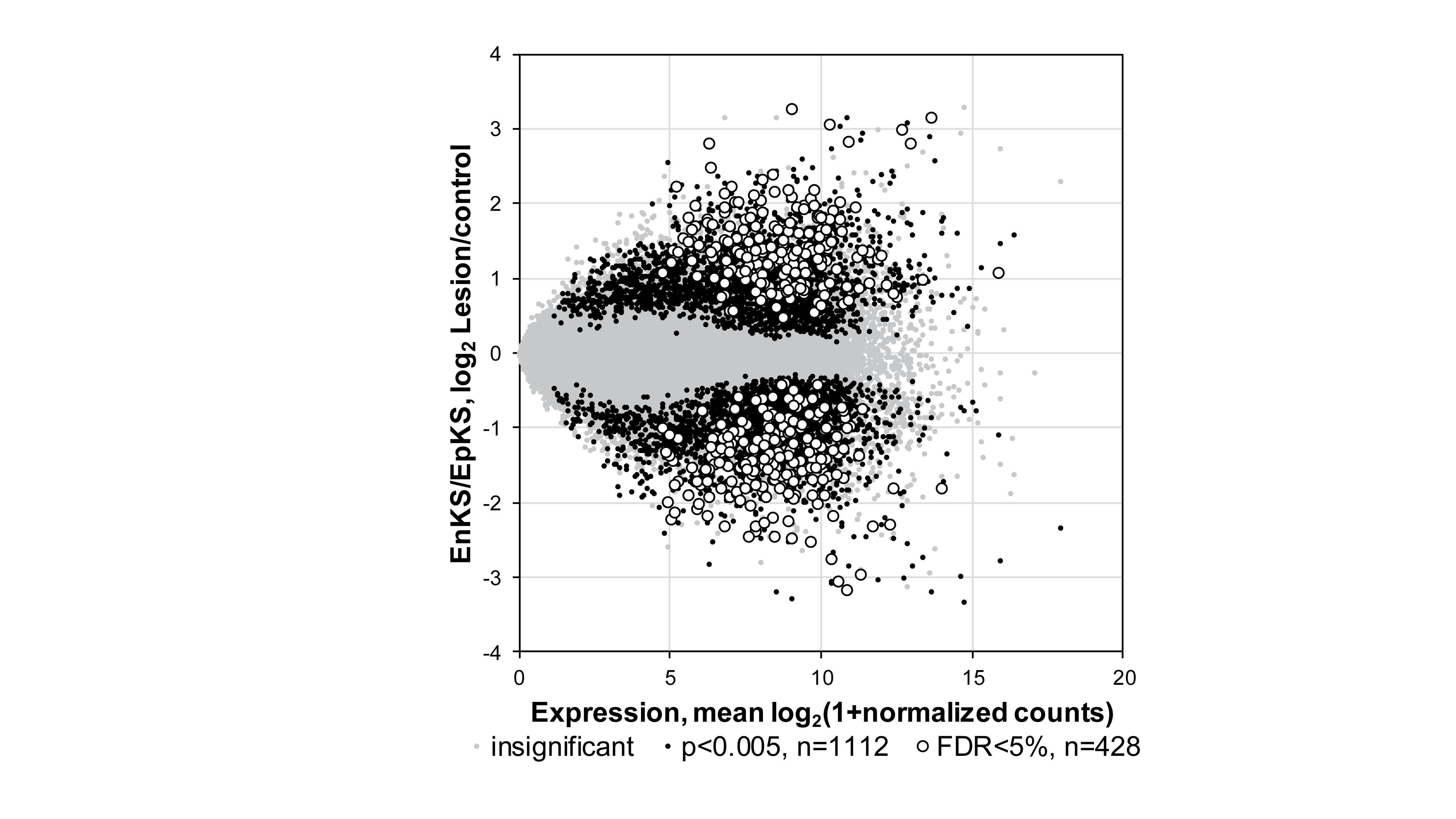

Supplement: S3 Fig — (TIF) [file ppat.1008681.s003.tif]

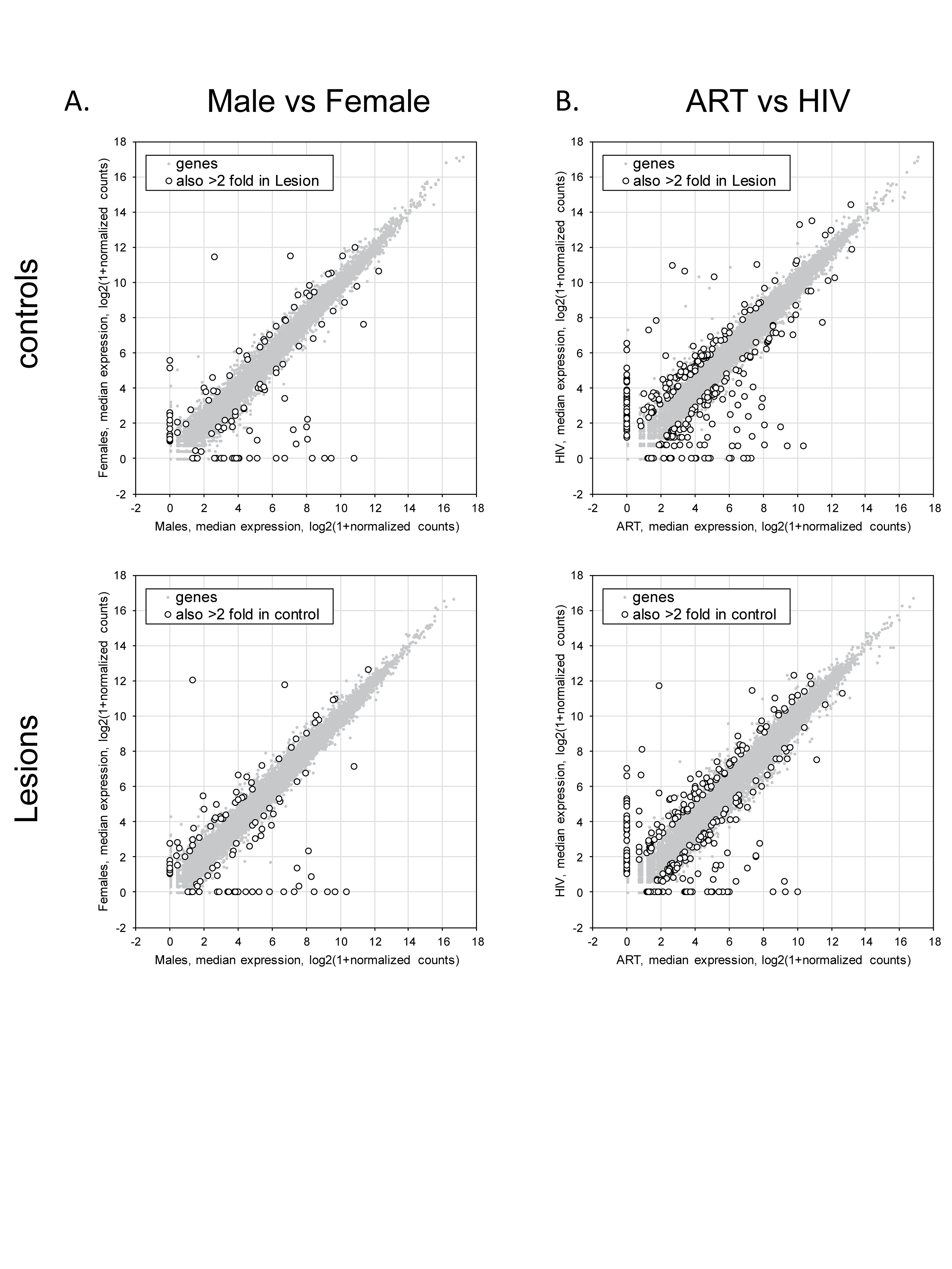

Supplement: S4 Fig — (A) Comparison of absolute normalized expression in controls and lesions between male and female. (B) Comparison of absolute normalized expression in controls and lesions between ART-treated and HIV (ART-naive) patients. (TIF) [file ppat.1008681.s004.tif]

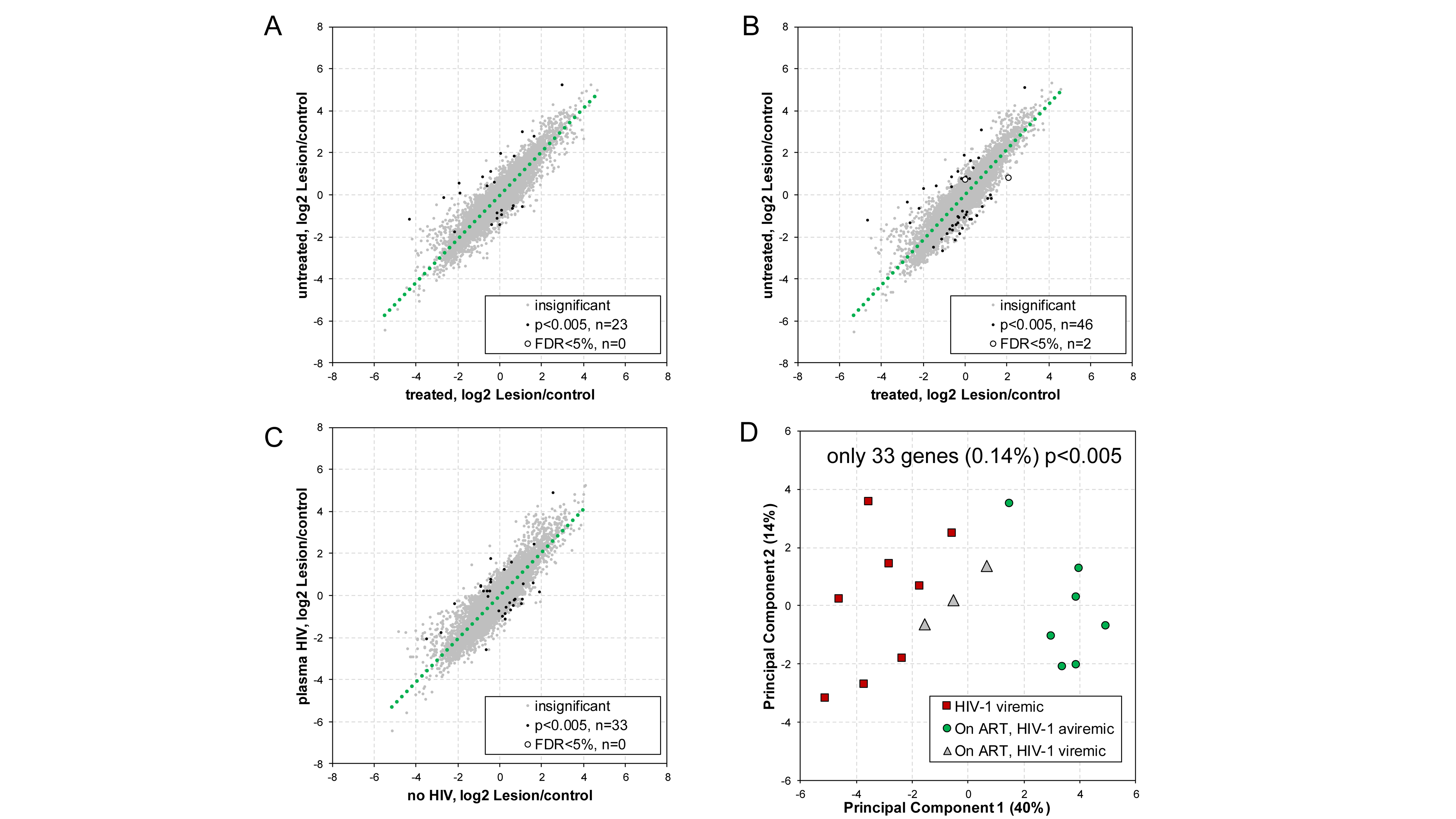

Supplement: S5 Fig — (A) A scatter plot comparing transcriptome profiles between EpKS patients treated with ART for more one week (N = 10) and those who were ART naïve or treated for less than a week (N = 8). Green dotted line denotes regression line. Grey dots denote genes whose expression was not significantly different between EpKS patients treated with Art for >1 week and those who are ART naïve of treated for <1 week. Black dots denote genes whose expression was significantly different between EpKS patients treated with Art for >1 week and those who are ART naïve of treated for <1 week at P = 0.005 (N = 23) and unfilled circles denote genes whose expression was significantly different between EpKS patients treated with Art for >1 week and those who are ART naïve of treated for <1 week at FDR<5% (N = 0). (B) A scatter plot comparing transcriptome profiles between EpKS patients treated with ART for more one month (N = 9) and those who were ART naïve or treated for less than a month (N = 9). Green dotted line denotes regression line. Grey dots denote genes whose expression was not significantly different between EpKS patients treated with Art for >1 month and those who are ART naïve of treated for <1 month. Black dots denote genes whose expression was significantly different between EpKS patients treated with Art for >1 month and those who are ART naïve of treated for <1 month at P = 0.005 (N = 46) and unfilled circles denote genes whose expression was significantly different between EpKS patients treated with Art for >1 month and those who are ART naïve of treated for <1 month at FDR<5% (N = 2). (C) A scatter plot comparing transcriptome profiles between EpKS patients with detectable plasma HIV-1 viral load (viremic) (N = 11) and undetectable plasma HIV-1 viral load (aviremic) (N = 7). Green dotted line denotes regression line. Grey dots denote genes whose expression was not significantly different between HIV-1 viremic and aviremic EpKS patients. Black dots denote genes whose expres [file ppat.1008681.s005.tif]
