# Supplementary material for: Comparative transcriptome analysis of endemic and epidemic Kaposi’s sarcoma (KS) lesions and the secondary role of HIV-1 in KS pathogenesis
Source: PLoS Pathog. 2020 Jul 24;16(7):e1008681. doi: 10.1371/journal.ppat.1008681 (PMC7406108; doi:10.1371/journal.ppat.1008681)
Supplement: S1 Table — EnKS–Endemic Kaposi’s sarcoma, EpKS–Epidemic Kaposi’s sarcoma. (DOCX) [file ppat.1008681.s006.docx]

| **HLA Super-type** | **Frequency** | | |
| --- | --- | --- | --- |
|  | **Healthy donors** | **EnKS** | **EpKS** |
| A01 | 3 | 4 | 6 |
| A02 | 1 | 4 | 9 |
| A03 | 1 | 0 | 6 |
| A24 | 1 | 1 | 8 |
| A01/A24 | 0 | 2 | 1 |
| A01/A03 | 0 | 0 | 1 |
| B58 | 0 | 1 | 1 |
| B44 | 0 | 1 | 0 |
| B27 | 2 | 2 | 3 |
| B07 | 2 | 5 | 12 |
| B58 | 1 | 2 | 6 |
| B08 | 0 | 0 | 2 |
| Unclassified | 0 | 1 | 2 |
