# Supplementary material for: Comparative transcriptome analysis of endemic and epidemic Kaposi’s sarcoma (KS) lesions and the secondary role of HIV-1 in KS pathogenesis
Source: PLoS Pathog. 2020 Jul 24;16(7):e1008681. doi: 10.1371/journal.ppat.1008681 (PMC7406108; doi:10.1371/journal.ppat.1008681)
Supplement: S2 Table — L–Lesion, C–Control, N–Normal/Healthy, KSHV–Kaposi’s sarcoma-associated herpesvirus, HIV-1 –Human Immunodeficiency virus type 1 (DOCX) [file ppat.1008681.s007.docx]

| **Sample** | **Type** | **Aligned to human genome** | **%** | **Aligned to exons** | **%** | **KSHV reads** | **HIV-1 reads** |
| --- | --- | --- | --- | --- | --- | --- | --- |
| 3212 | N | 16,203,264 | 74% | 8,534,963 | 39% | 2 | 0 |
| 3214 | N | 12,942,249 | 62% | 6,765,416 | 32% | 30 | 0 |
| 3215 | N | 17,165,729 | 77% | 9,451,684 | 42% | 0 | 0 |
| 3111 | C | 19,712,438 | 77% | 10,573,186 | 41% | 8 | 0 |
| 3111 | L | 20,333,290 | 75% | 11,804,691 | 44% | 16,278 | 0 |
| 3124 | C | 17,643,613 | 76% | 9,594,565 | 41% | 0 | 0 |
| 3124 | L | 20,922,117 | 76% | 12,136,413 | 44% | 14,212 | 0 |
| 3135 | C | 14,671,781 | 70% | 7,700,580 | 37% | 25 | 0 |
| 3135 | L | 25,618,227 | 75% | 15,275,642 | 45% | 31,037 | 0 |
| 3136 | C | 15,656,184 | 77% | 8,339,846 | 41% | 0 | 0 |
| 3136 | L | 17,448,164 | 76% | 10,091,860 | 44% | 3,624 | 0 |
| 3139 | C | 19,907,748 | 72% | 11,483,855 | 41% | 765 | 0 |
| 3139 | L | 20,062,720 | 70% | 11,930,178 | 42% | 8,818 | 0 |
| 3140 | C | 11,728,595 | 67% | 5,516,681 | 32% | 10 | 0 |
| 3140 | L | 24,451,254 | 75% | 14,426,893 | 44% | 7,497 | 0 |
| 3129 | C | 18,076,213 | 75% | 9,309,349 | 38% | 5 | 0 |
| 3129 | L | 22,898,552 | 73% | 13,231,001 | 42% | 2,702 | 0 |
| 3122 | C | 20,014,697 | 75% | 11,304,528 | 42% | 1,417 | 0 |
| 3122 | L | 21,857,023 | 71% | 12,927,067 | 42% | 6,798 | 0 |
| 3128 | C | 20,995,075 | 74% | 12,160,646 | 43% | 207 | 0 |
| 3128 | L | 27,076,222 | 75% | 16,216,999 | 45% | 14,364 | 0 |
| 3032 | C | 11,089,139 | 78% | 5,380,911 | 38% | 0 | 0 |
| 3032 | L | 12,942,152 | 81% | 6,596,422 | 41% | 718 | 0 |
| 3022 | C | 11,172,170 | 81% | 5,527,199 | 40% | 2 | 0 |
| 3022 | L | 12,622,243 | 82% | 6,742,469 | 44% | 1,650 | 0 |
| 21083 | C | 11,639,391 | 79% | 5,729,612 | 39% | 60 | 0 |
| 21083 | L | 13,254,001 | 83% | 6,998,716 | 44% | 3,441 | 0 |
| 035 | C | 26,397,477 | 76% | 14,205,852 | 41% | 0 | 0 |
| 035 | L | 23,574,467 | 69% | 14,327,393 | 42% | 3,278 | 12 |
| 034 | C | 28,051,240 | 77% | 15,406,695 | 42% | 1 | 1 |
| 034 | L | 26,361,549 | 74% | 15,387,012 | 43% | 3,315 | 6 |
| 026 | C | 25,507,023 | 73% | 15,078,502 | 43% | 0 | 0 |
| 026 | L | 26,645,546 | 73% | 15,385,664 | 42% | 1,437 | 2 |
| 025 | C | 25,830,904 | 76% | 12,854,206 | 38% | 5 | 0 |
| 025 | L | 26,084,914 | 76% | 15,637,358 | 45% | 17,865 | 0 |
| 023 | C | 26,559,463 | 76% | 14,417,844 | 41% | 1 | 3 |
| 023 | L | 25,057,539 | 74% | 14,720,002 | 43% | 18,183 | 11 |
| 038 | C | 27,124,931 | 76% | 15,076,489 | 42% | 2 | 0 |
| 038 | L | 29,273,901 | 75% | 17,821,720 | 45% | 18,866 | 0 |
| 21196 | C | 28,690,725 | 79% | 15,808,859 | 44% | 21 | 0 |
| 21196 | L | 27,303,103 | 77% | 15,080,446 | 42% | 7,303 | 0 |
| 21199 | C | 26,949,290 | 79% | 14,552,377 | 43% | 0 | 0 |
| 21199 | L | 29,421,653 | 77% | 16,735,357 | 44% | 2,975 | 0 |
| 21214 | C | 29,076,407 | 79% | 15,390,716 | 42% | 1 | 0 |
| 21214 | L | 33,945,515 | 77% | 19,986,850 | 45% | 10,219 | 4 |
| 21221 | C | 28,840,087 | 78% | 15,623,416 | 42% | 0 | 0 |
| 21221 | L | 28,204,014 | 77% | 16,573,348 | 45% | 21,076 | 0 |
| 032 | C | 28,466,918 | 79% | 15,370,747 | 43% | 0 | 0 |
| 032 | L | 33,507,327 | 76% | 19,589,442 | 44% | 7,563 | 0 |
| 037 | C | 27,033,582 | 78% | 14,987,641 | 43% | 650 | 1 |
| 037 | L | 29,707,575 | 75% | 17,693,581 | 45% | 3,264 | 0 |
